# HLA-B locus products resist degradation by the human cytomegalovirus immunoevasin US11

**DOI:** 10.1371/journal.ppat.1008040

**Published:** 2019-09-17

**Authors:** Cosima Zimmermann, Daniel Kowalewski, Liane Bauersfeld, Andreas Hildenbrand, Carolin Gerke, Magdalena Schwarzmüller, Vu Thuy Khanh Le-Trilling, Stefan Stevanovic, Hartmut Hengel, Frank Momburg, Anne Halenius

**Affiliations:** 1 Institute of Virology, Medical Center University of Freiburg, Freiburg, Germany; 2 Faculty of Medicine, University of Freiburg, Freiburg, Germany; 3 Department of Immunology, Interfaculty Institute for Cell Biology, University of Tübingen, Tübingen, Germany; 4 Spemann Graduate School of Biology and Medicine (SGBM), University of Freiburg, Freiburg, Germany; 5 Faculty of Biology, University of Freiburg, Freiburg, Germany; 6 Institute for Virology, University Duisburg-Essen, Essen, Germany; 7 Clinical Cooperation Unit Applied Tumor Immunity, Antigen Presentation and T/NK Cell Activation Group, German Cancer Research Center, Heidelberg, Germany; University of Wisconsin-Madison, UNITED STATES

## Abstract

To escape CD8+ T-cell immunity, human cytomegalovirus (HCMV) US11 redirects MHC-I for rapid ER-associated proteolytic degradation (ERAD). In humans, classical MHC-I molecules are encoded by the highly polymorphic HLA-A, -B and -C gene loci. While HLA-C resists US11 degradation, the specificity for HLA-A and HLA-B products has not been systematically studied. In this study we analyzed the MHC-I peptide ligands in HCMV-infected cells. A US11-dependent loss of HLA-A ligands was observed, but not of HLA-B. We revealed a general ability of HLA-B to assemble with β_2_m and exit from the ER in the presence of US11. Surprisingly, a low-complexity region between the signal peptide sequence and the Ig-like domain of US11, was necessary to form a stable interaction with assembled MHC-I and, moreover, this region was also responsible for changing the pool of HLA-B ligands. Our data suggest a two-pronged strategy by US11 to escape CD8+ T-cell immunity, firstly, by degrading HLA-A molecules, and secondly, by manipulating the HLA-B ligandome.

## Author summary

The human immune system can cover the presentation of a wide array of pathogen derived antigens owing to the three extraordinary polymorphic MHC class I (MHC-I) gene loci, called HLA-A, -B and -C in humans. Studying the HLA peptide ligands of human cytomegalovirus (HCMV) infected cells, we realized that the HCMV encoded glycoprotein US11 targeted different HLA gene products in distinct manners. More than 20 years ago the first HCMV encoded MHC-I inhibitors were identified, including US11, targeting MHC-I for proteasomal degradation. Here, we describe that the prime target for US11-mediated degradation is HLA-A, whereas HLA-B can resist degradation. Our further mechanistic analysis revealed that US11 uses various domains for distinct functions. Remarkably, the ability of US11 to interact with assembled MHC-I and modify peptide loading of degradation-resistant HLA-B was dependent on a low-complexity region (LCR) located between the signal peptide and the immunoglobulin-like domain of US11. To redirect MHC-I for proteasomal degradation the LCR was dispensable. These findings now raise the intriguing question why US11 has evolved to target HLA-A and -B differentially. Possibly, HLA-B molecules are spared in order to dampen NK cell attack against infected cells.

## Introduction

Human cytomegalovirus (HCMV) represents a prototypic β-herpesvirus persisting throughout life in its host with periodic phases of latency and reactivation of productive infection. Despite rare cases of clinical disease in healthy individuals, HCMV has a permanent impact on immune cells, e.g. resulting in the extraordinary expansion of both CD8+ memory T-cells and memory-like NK cells [1, 2]. As demonstrated in CMV animal models, protection from CMV disease is strongly dependent on MHC class I (MHC-I) restricted CD8+ T-cell responses [3].

MHC-I molecules are dimers formed by the membrane attached heavy chain (HC) and the soluble beta-2-microglobulin (β_2_m). Upon assembly in the ER the heterodimeric MHC-I molecule is recruited to the peptide loading complex (PLC), composed of the transporter associated with antigen processing (TAP) and the chaperones tapasin, ERp57 and calreticulin [4, 5]. In the course of loading an optimal peptide, the trimolecular MHC-I complex is released for transport through the secretory pathway and surface expression.

The *US6* gene family region of HCMV encodes for several immunoevasins that target the MHC-I antigen presentation pathway at different stages of the protracted HCMV replication cycle [6]. US3 blocks maturation of MHC-I and interferes with tapasin function, US2 and US11 target MHC-I for rapid proteasomal degradation, and US6 inhibits peptide translocation by TAP [7–13].

Of the reported HCMV encoded MHC-I inhibitors, so far a crystal structure exists only for US2 in complex with HLA-A*02:01 [14]. Similar to US2, US11 binds to MHC-I with its ectodomain, but the contact site on MHC-I has not been defined [15, 16]. The lack of insight into the contacts sites is mirrored by the poor understanding of the HLA allotype specificity of US11. Whereas varying effects of US11 on HLA-B and -C allotypes have been reported [17–20], consistent downregulation of HLA-A allotypes was observed [19–21].

HLA-A*02:01 has been an instrumental substrate to elucidate ER associated degradation (ERAD) pathways activated by US11. Upon binding to MHC-I, a glutamine in the transmembrane segment of US11 recruits Derlin-1 [15, 22]. Derlin-1 is a crucial component of an ERAD pathway also including the recently identified E3 ligase TMEM129 and the E2 ligase Ube2J2, required for ubiquitination and subsequent degradation of MHC-I [23, 24]. Whereas US11 itself is not degraded by this pathway, low MHC-I expression exposes US11 to an alternative degradation pathway including HRD1 and SEL1L [24, 25].

The genes of the *US6* family most likely evolved in a cytomegalovirus ancestor prior to the split of Old World monkeys and hominoids, as homologs of these genes are found also in chimpanzee and rhesus CMV (rhCMV) [26, 27]. In the context of a rhCMV vaccine vector [28], in addition to degradation of MHC-I, it was observed that the rhCMV US11 homolog Rh189 is able to suppress the CD8+ T-cell responses to canonical epitopes [29]. Although the data are not formally published, Früh and Picker mention in a recent overview publication that substitution of Rh189 with HCMV US11 in a rhCMV mutant maintains the ability of the virus to block canonical CD8+ T-cell responses [28], suggesting that in addition to MHC-I degradation, US11 proteins execute further manipulation of MHC-I antigen presentation.

When studying the MHC-I ligandome in HCMV-infected cells, we noticed a striking differential effect of US11 on HLA class I locus products. Ligands from HLA-B and -C molecules remained largely unaltered in the presence of US11, while HLA-A ligands were efficiently eliminated. Further investigations reported here revealed that US11 targets HLA-B by manipulating the quality of the peptide ligands. This illustrates the specific role of a single MHC-I immunoevasin that not randomly degrades MHC-I molecules, but has evolved to exert HLA locus-specific functions.

## Results

### US11 diminishes HLA-A but not HLA-B specific peptides in HCMV-infected MRC-5 fibroblasts

**Fig 1:**
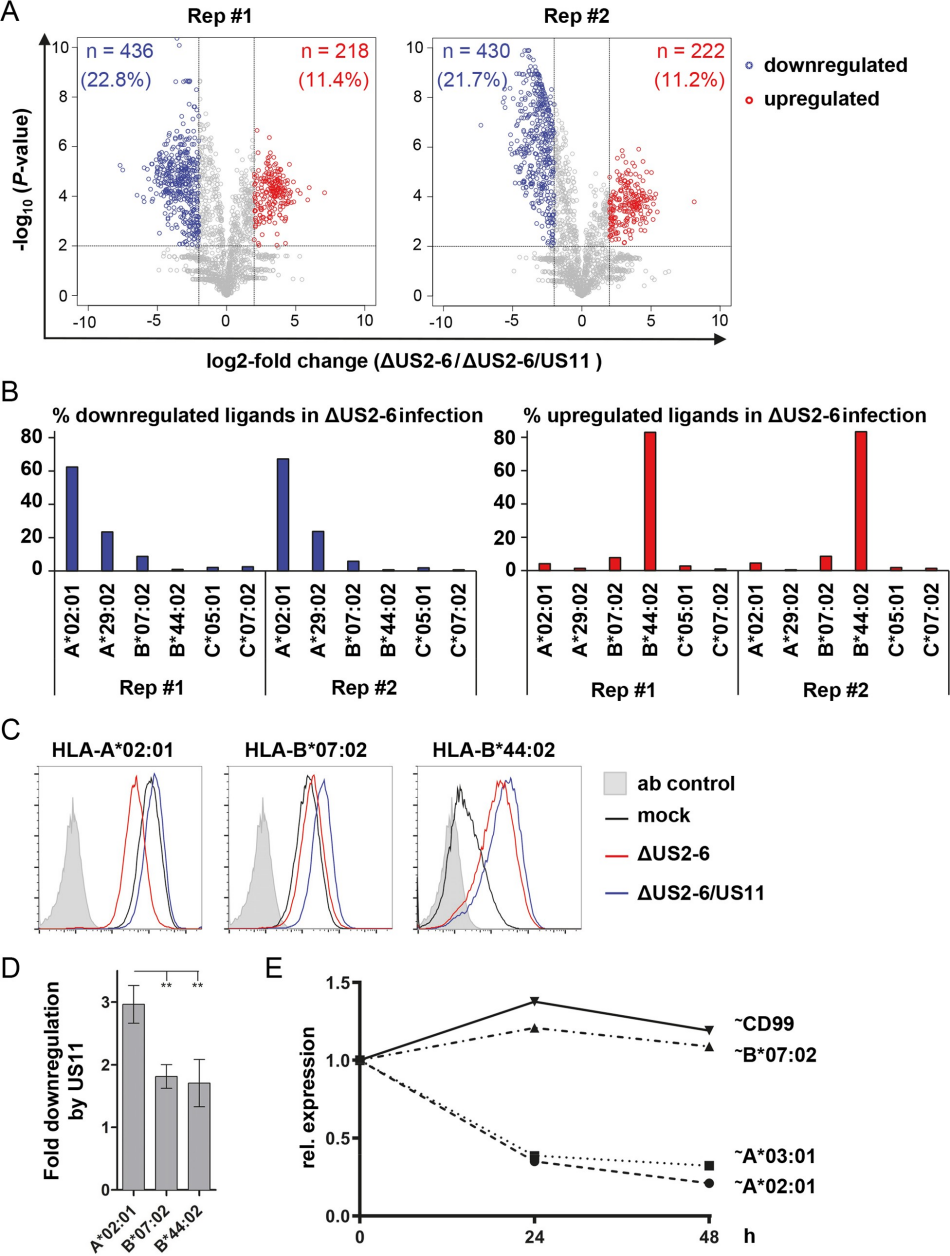
Changes in the relative abundance of HLA class I ligands in HCMV-infected fibroblasts. MRC-5 cells infected with ΔUS2-6 or ΔUS2-6/US11 HCMV mutants at a multiplicity of infection (MOI) of 5 were collected at 48 h post-infection and MHC-I molecules were immunoprecipitated using the mAb W6/32. Peptide ligands were eluted and analyzed by mass spectrometry. **(A)** Volcano plots of changes in the relative abundances of HLA ligands in the two samples (Rep. #1 and #2). Each dot represents a specific HLA ligand. Log2-fold-changes of their abundance in US11+ compared to US11- infection are indicated on the x-axis, the corresponding significance levels after Benjamini-Hochberg correction are given on the y-axis. HLA ligands showing significant up- or downregulation (>4-fold change in abundance with p<0.01) are highlighted in red and blue, respectively. The numbers and percentages of significantly modulated ligands are specified in the corresponding quadrants. The reproducibility of HLA peptidome analysis was assessed by plotting HLA-I peptide abundances in biological replicates of HCMV infected MRC-5 cells (S2A Fig) **(B)** Distribution of HLA restrictions among peptides significantly down-regulated (blue) or up-regulated (red), as identified in A. **(C)** MRC-5 cells were mock-treated or infected with ΔUS2-6 or ΔUS2-6/US11 deletion mutants at an MOI of 5. At 48 h post-infection MHC-I cell surface expression was analyzed by flow cytometry with mAbs as indicated. **(D)** Quantification of fold downregulation by US11 shown in (C), as ratio of MFI measured in ΔUS2-6/US11-infected cells relative to Δ*US2-6*. Error bars show SEM for at least three independent experiments. Statistical analyses were performed applying one-way analysis of variance (ANOVA) followed by a Tuckey’s multiple comparisons method for all pairwise differences of means. **(E)** HeLa cells stably expressing HA-tagged (~) HLA-A*02:01, A*03:01, B*07:02 or CD99 molecules were transduced with lentiviruses encoding US11 in front of an IRES-EGFP sequence. At 0, 24 and 48 h post-transduction the cells were analyzed by flow cytometry using an anti-HA mAb. The MFI in EGFP^+^ cells relative to MFI at 0 h post transduction is depicted. Two independent biological replicates of the experiment with similar outcomes were performed.

To gain deeper insight into the identity and amount of HCMV peptides presented by MHC-I we undertook MHC-I ligandome analysis. MRC-5 fibroblasts were infected with HCMV AD169VarL strain derived mutants lacking either the MHC-I inhibitor region *US2-US6* (ΔUS2-6) or *US2-US6* plus *US11* (ΔUS2-6/US11), leaving the *US7-US10* region intact as demonstrated by the expression of the neighboring gene *US10* in ΔUS2-6/US11 infected cells (S1 Fig). At 48 h post-infection cells were harvested and MHC-I complexes were isolated by the pan*-*MHC*-*I-reactive monoclonal antibody W6/32. Identification and relative quantification of HLA ligands was performed by LC-MS/MS. Much to our surprise, a clear difference in the ability of US11 to target HLA-A and HLA-B molecules was observed. About 90% of all peptide ligands significantly down-regulated by US11 (ca 22% of total pool; Fig 1A, blue dots) was derived from HLA-A*02:01 and A*29:02 molecules (Fig 1B, left panel). Unexpectedly, some peptides (ca 11% of the total pool) were found to be increased in cells infected with the ΔUS2-6 mutant (Fig 1A, red dots). The majority (ca 80%) of these peptides could be attributed to HLA-B*44:02 (Fig 1B, right panel). No major changes were observed for the ligandome of HLA-B*07:02. To reassure that our observations were not distorted by ligands from uninfected cells possibly present in the culture, we analyzed the anchor residues of identified HCMV-derived peptides to predict their HLA-I specificities. The distribution of these viral peptides showed the same HLA-I pattern as the total ligandome, but more pronounced; US11 almost completely abolished viral ligands of HLA-A, but not of HLA-B. Again, HLA-B*44:02 ligands increased in the presence of US11 (S2B Fig).

Since the protocol for ligandome analysis does not differentiate between surface and intracellular MHC-I molecules we next conducted flow cytometry analysis of MRC-5 fibroblasts mock treated or infected with the ΔUS2-6 or ΔUS2-6/US11 deletion viruses, to determine the surface levels of HLA-A and HLA-B using available specific antibodies for HLA-A*02:01, B*07:02 and B*44:02. Whereas less than two-fold reduction for HLA-B surface expression was observed (Fig 1C and 1D), HLA-A*02:01 was reduced 3-fold in the presence of US11 as compared to infection with the virus lacking US11. Therefore, also on the surface on HCMV-infected fibroblasts, a stronger effect of US11 was measured for the HLA-A allotype A*02:01 than for the HLA-B allotypes B*07:02 and B*44:02. Stronger regulation of HLA-A*02 was also observed on ARPE19 epithelial cells infected with the TB40 derived ΔUS2-6 BAC virus. Compared to mock-treated cells HLA-A*02 was downregulated 5-fold and HLA-B*07 1.5-fold (S1E Fig), indicating that different regulation of HLA-A*02:01 and HLA-B*07:02 by US11 is not a fibroblast adapted function of HCMV.

We wondered whether the strong resistance of HLA-B*07:02 would also hold true in the context of non-infected cells and therefore N-terminally HA-tagged (HA-tagged molecules are indicated with ~ in all following figures) HLA-A*02:01, A*03:01, B*07:02 and CD99 were cloned into a lentiviral vector. HeLa cells were first transduced and selected to express the MHC-I molecules or the control protein CD99 and subsequently transduced with a lentivirus encoding US11 in front of an IRES and *EGFP* sequence. Cell surface expression of HA-tagged MHC-I and CD99 was analyzed on EGFP positive cells in comparison to EGFP negative cells at 0, 24 and 48 h after transduction. Remarkably, in this context, similarly to the control protein CD99, HLA-B*07:02 appeared completely unaffected by US11, but not HLA-A*02:01 and A*03:01 (Fig 1E), demonstrating that resistance of HLA-B*07:02 against US11 is independent of other viral or virally induced proteins. In infected cells, US11 may work in concert with other proteins and could explain the stronger effect of US11 on HLA-B in infected MRC-5 cells. HCMV also reorganizes the secretory pathway in infected cells [30], which could further influence trafficking and surface expression of MHC-I allotypes in the presence of US11.

### HLA-B allotypes can escape US11-mediated degradation

**Fig 2:**
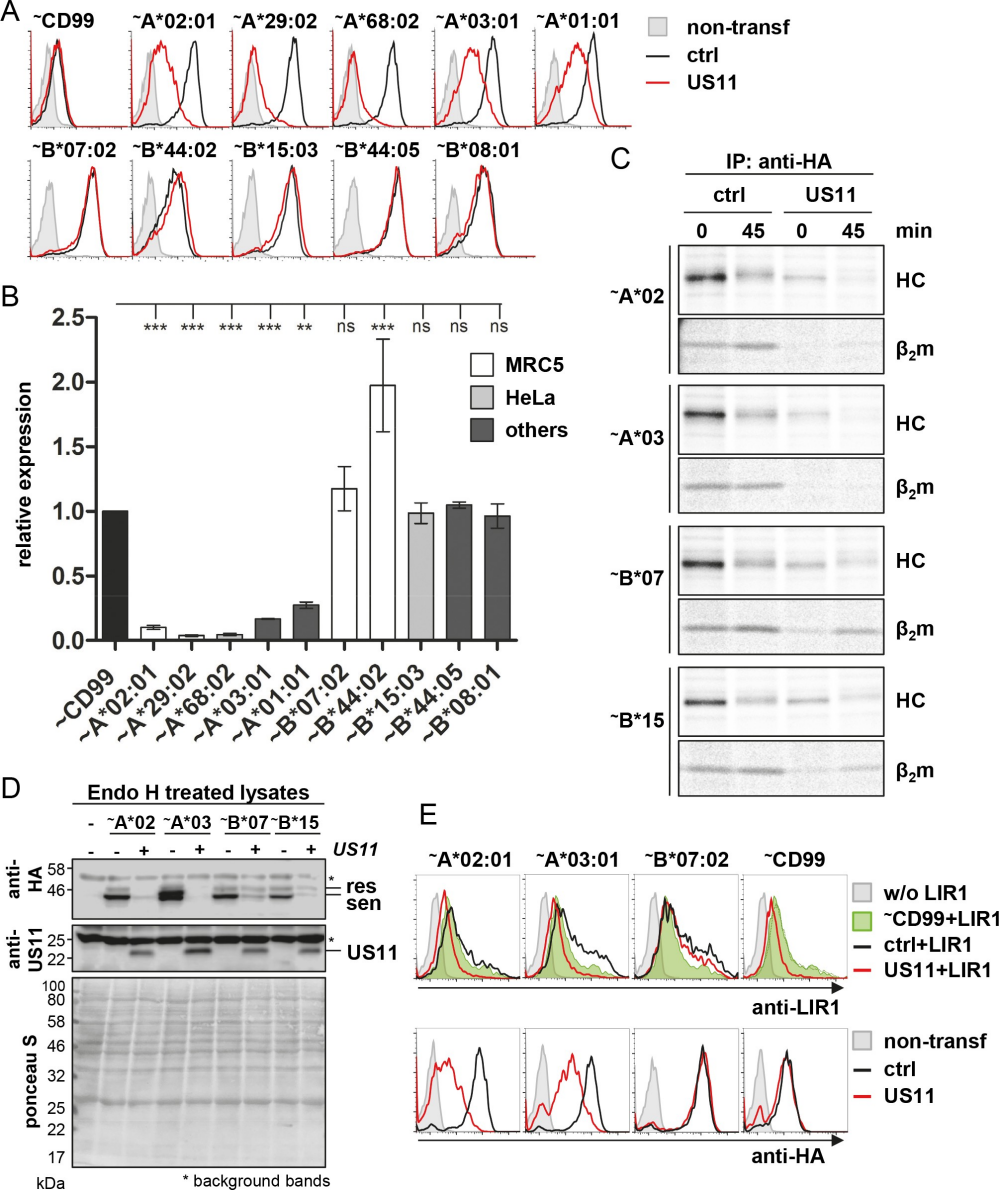
HLA locus specific downregulation by US11. HeLa cells were transiently co-transfected with US11 or a control pIRES-EGFP plasmid (CMV major IE promoter) together with the indicated HA-tagged (~) HLA molecules expressed from the pUC-IP vector (SFFV U3 promoter). **(A)** At 20 h post-transfection HLA-I cell surface expression of EGFP positive cells was measured by flow cytometry using anti-HA mAbs. **(B)** The HLA-I expression from (A) was defined as ratio of the MFI in US11 expressing cells compared to control cells, the value of which was normalized to the downregulation of a control molecule (HA-CD99). Bars represent normalized mean values ± SEM from three independent experiments. Statistical analyses were performed to compare HLA-A or -B alleles among themselves, applying one-way ANOVA followed by a Tuckey’s multiple comparisons method for all pairwise differences of means. Endogenous HLA-I expressed in MRC-5 or HeLa cells are indicated. **(C)** At 20 h post-transfection cells were labeled with [^35^S]-Met/Cys for 30 min and chased for 0 or 45 min and an immunoprecipitation was performed using anti-HA mAb. An uncropped autoradiography is depicted in S3 Fig. **(D)** Whole cell lysates were prepared and digested with EndoH prior to analysis by Western blot with antibodies as indicated. Equal loading of lysates was controlled by Ponceau S staining. **(E)** Cells were analyzed as described in A. In addition, the cells were incubated with LIR1-Fc. In the upper panel binding of LIR1-Fc to the CD99/ctrl transfected cells is shown in green. Representatives of two independent biological replicates with similar outcomes are shown.

To gain insight into the breadth of the resistance of HLA-B locus products against US11, we cloned various HLA-A and -B sequences with an N-terminal HA-tag und analyzed the cell surface level in the absence and presence of US11 after transient transfection of HeLa cells. Usage of the CMV major IE promoter for MHC-I expression leads to only low level of MHC-I regulation (probably due to overload of the ER and insufficient levels of ERAD-associated proteins required for US11 function) and we therefore proceeded with a different vector harboring a less potent promoter (spleen focus-forming virus (SFFV) U3 promoter) to be able to measure an adequate level of US11-mediated downregulation. Using this approach, all HLA-A allotypes were strongly downregulated by US11 (Fig 2A and 2B), while no significant reduction of HLA-B cell surface expression was noted. However, HLA-B*44:02, from which more diverse peptide ligands were eluted in the presence of US11 in infected cells (Fig 1B, right panel), displayed an induced level of surface expression in US11-expressing cells. Altogether, this suggests that the observed resistance towards US11-mediated downregulation is a general feature of HLA-B locus products.

Next, pulse-chase experiments were conducted to measure the stability of MHC-I molecules in the absence and presence of US11. As expected, a clear destabilization of HLA-A*02:01 and A*03:01 could be observed. Surprisingly, also the stability of HLA-B HCs was strongly reduced in the presence of US11 (Fig 2C and S4 Fig, using shorter labeling and chase times). However, in contrast to HLA-A, HLA-B allotypes were able to dimerize with β_2_m in the presence of US11 and maturation of these molecules could be observed as a slightly slower migrating HC band (likely due to glycan modifications) at 45 min of chase (Fig 2C). This observation suggests, that different from HLA-A, HLA-B molecules are able to exit the ER and accumulate at the cell surface in the presence of US11. We therefore analyzed the steady-state level of EndoH (Endoglycosidase H; digests Asn-linked glycans that have not been subjected to modification in the Golgi apparatus) resistant molecules by Western blot and indeed observed that although the total level of HLA-B was reduced in the presence of US11, the EndoH resistant molecules were largely unchanged compared to control cells. This was not the case for HLA-A, as a complete loss of EndoH resistant molecules was observed (Fig 2D). We asked whether HLA-B surface expression in the presence of US11 could be more readily recognized by an inhibitory MHC-I receptor such as LIR1. Indeed, LIR1-Fc incubated with HeLa cells treated as described above showed a clear binding to HLA-B*07:02 expressing cells despite co-transfection of US11, whereas this was not the case for HLA*02:01 and -A*03:01 expressing cells (Fig 2E).

In conclusion, HLA-A and also HLA-B molecules are targets for US11-mediated degradation. However, different from HLA-A, a fraction of the HLA-B molecules can escape degradation and be expressed on the cell surface.

### US11 binds both HLA-A and HLA-B molecules

**Fig 3:**
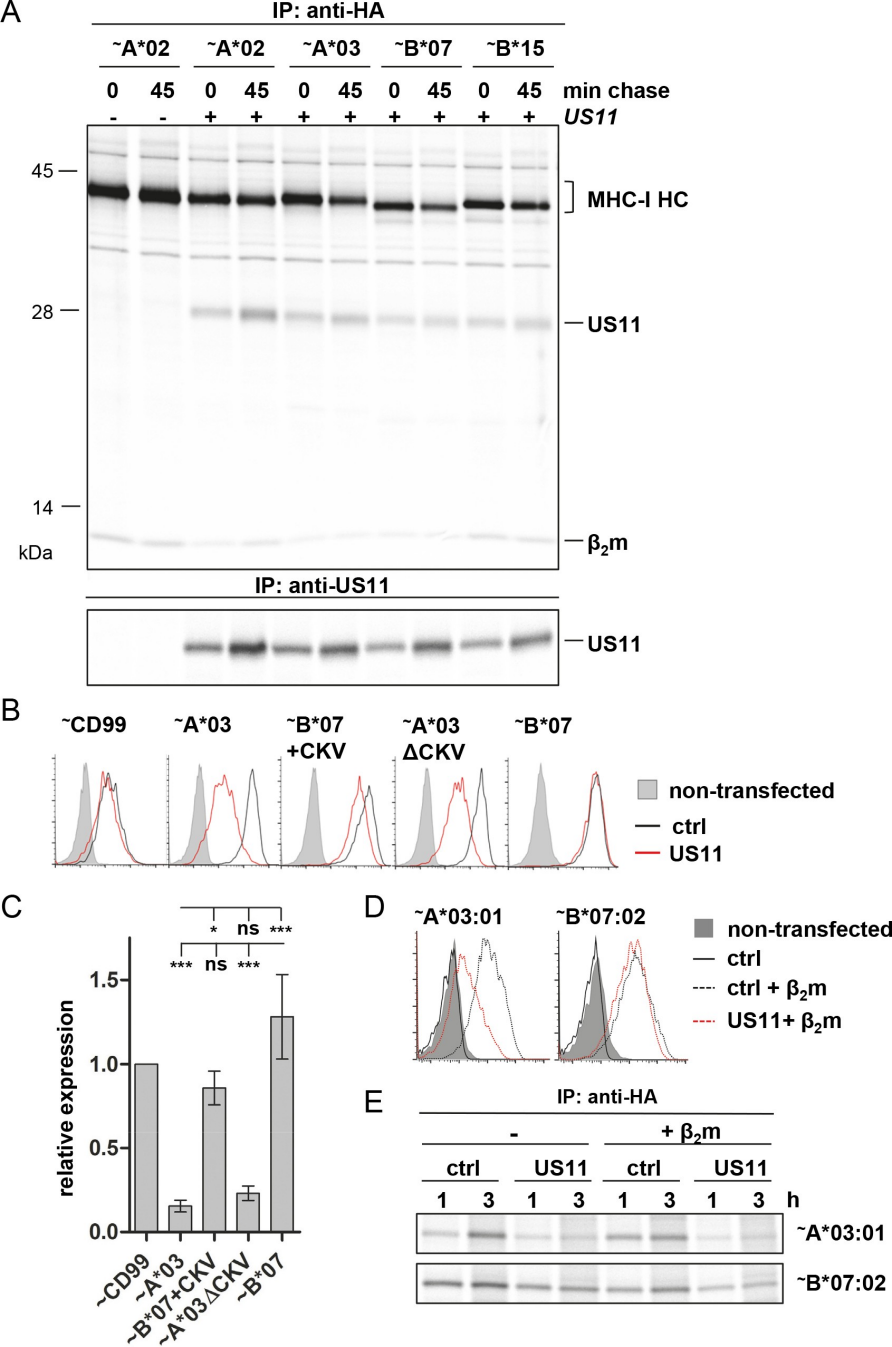
Analysis of factors that could affect HLA-B resistance against US11. **(A)** HeLa cells were transiently co-transfected with US11 or a ctrl pIRES-EGFP plasmid together with indicated HA-tagged (~) HLA in pIRES-EGFP (CMV major IE promoter). At 20 h post-transfection cells were labeled with [^35^S]-Met/Cys for 30 min and chased for 0 or 45 min and a co-immunoprecipitation experiment was performed using anti-HA mAb or anti-US11 antiserum. A complete autoradiography is depicted in S5 Fig. A representative of two independent biological replicates with similar outcomes is shown. **(B-C)** HeLa cells were transiently co-transfected with US11 or a ctrl pIRES-EGFP plasmid together with indicated HA-tagged (~) MHC-I molecules and mutants encoded by the pUC-IP vector. At 20 h post-transfection flow cytometry and statistical analysis were performed as described in Fig 2A and 2B. **(D)** β_2_m-deficient FO-1 cells were co-transfected with US11 or a control pIRES-EGFP plasmid together with indicated HA-tagged (~) HLA alleles encoded by the pUC-IP vector. At 20 h post-transfection cell surface expression of EGFP positive cells was measured by flow cytometry using anti-HA mAbs. **(E)** FO-1 cells were transfected as described in (D). At 20 h post-transfection cells were labeled with [^35^S]-Met/Cys for 1 or 3 h and an immunoprecipitation was performed using anti-HA antibodies. A representative of two independent biological replicates with similar outcomes is shown. A complete autoradiography is depicted in S6 Fig.

In the pulse-chase experiment we observed an apparent co-immunoprecipitation of US11 with all studied types of MHC-I molecules (S3 and S4 Figs). This suggested that although HLA-B molecules escape downregulation by US11, they are still bound by US11 efficiently. To depict this more clearly, we took advantage of the fact that MHC-I molecules expressed transiently from a strong promoter (CMV IE promoter) remain largely stable in the presence of US11. Under these conditions we assessed binding of US11 to HLA-A*02:01, A*03:01, B*07:02 and B*15:03 (Fig 3A). With US11 and MHC-I being strongly overexpressed at saturating levels, US11 bound similarly to all MHC-I molecules.

We next assessed the possibility that the cytosolic tail of HLA-A allotypes, which is three residues (CysLysVal) longer than that of HLA-B allotypes, could be decisive for differential US11 regulation. The C-terminal Val has previously been described to be important for US11-mediated degradation [31]. To this end, we constructed an HLA-A*03:01 mutant without these residues (A3ΔCKV) and compared it to the reciprocal HLA-B*07:02 mutant (B7+CKV). The residues CysLysVal had only small and non-significant effects on surface expression in US11 expressing cells as measured by flow cytometry (Fig 3B and 3C). Even though HLA-B*07 was more efficiently downregulated, when expressed with C-terminal CysLysVal residues, downregulation was significantly different from HLA-A*03:01. Therefore, the C-terminal CysLysVal residues are not the major determinant for the difference in US11-mediated regulation.

We next scrutinized the role of β_2_m in the process of US11-mediated degradation, since the HLA-B molecules HLA-B8 and -B5 were reported to possess a higher affinity for β_2_m compared to HLA-A1 and -A2 [32]. In FO-1 cells MHC-I is not expressed on the surface due to lack of β_2_m. Co-transfection of a plasmid encoding β_2_m rescued cell surface expression of MHC-I (Fig 3D). Similarly to HeLa cells, in FO-1 cells transfected with a β_2_m encoding plasmid, HLA-A*03:01 was downregulated by US11, whereas HLA-B*07:02 was not (Fig 3D). Also the stability of HLA-B*07:02 was higher compared to A*03:01, and this was not influenced by β_2_m (Fig 3E), implying that the resistance of HLA-B against US11 is conferred at the stage of unassembled HC.

In conclusion, although US11 binding to HLA-B is preserved, downregulation of HLA-B is much less efficient and this is only partly due to the shorter cytosolic tail of HLA-B alloforms.

### The N-terminal low-complexity region of US11 confers binding to MHC-I/β_2_m heterodimers and ER retention

**Fig 4:**
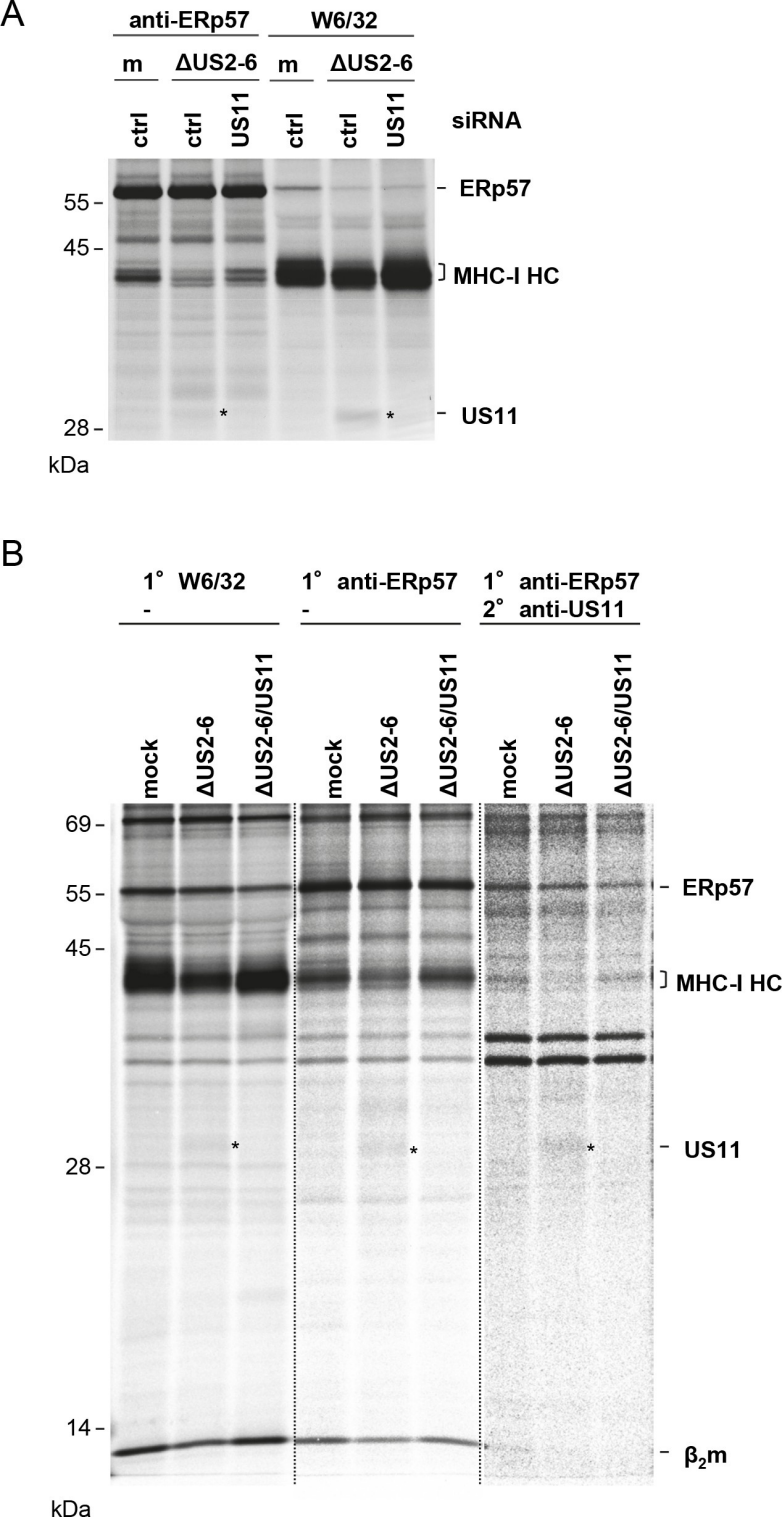
US11 co-immunoprecipitation with the PLC in HCMV-infected cells. (**A**) MRC-5 cells were treated with US11_1 (US11) or control (ctrl) siRNA (S7A Fig) 4 h prior to mock treatment (m) or infection with the ΔUS2-6 HCMV mutant at an MOI of 5. At 24 h post-infection cells were metabolically labeled with [^35^S]-Met/Cys for 2 h and an immunoprecipitation using anti-ERp57 or W6/32 mAbs was performed. **(B)** MRC-5 cells were mock treated or infected with ΔUS2-6 or ΔUS2-6/US11 HCMV mutants at an MOI of 5. At 24 h post-infection, cells were metabolically labeled with [^35^S]-Met/Cys for 2 h and an immunoprecipitation using W6/32 or anti-ERp57 antibodies was performed. In addition, anti-ERp57 recovered proteins were dissociated and re-immunoprecipitated using an anti-US11 antiserum. The gel image is displayed with various contrast and light conditions depending on the antibodies (marked by dotted lines) used. The image is displayed with same conditions for all parts in S7B Fig.

In our previous studies of the PLC composition in HCMV-infected cells [33], co-immunoprecipitation experiments suggested that US11 interacts with MHC-I as part of the the PLC. This was unexpected, as the concept for MHC-I targeting by US11 has been a rapid degradation facilitated by ERAD. However, in light of our observation, that HLA-B molecules display intrinsic resistance against US11-mediated degradation, we supposed that US11 pursues two different strategies when interacting with HLA-A and HLA-B, respectively. To re-investigate our earlier findings, MRC-5 fibroblasts were treated with siRNA targeting US11 or control siRNA and subsequently infected with the ΔUS2-6 HCMV deletion mutant. At 24 h post-infection a co-immunoprecipitation experiment was performed using W6/32 or anti-ERp57 antibodies. As expected, a band corresponding to the size of US11 was found in complex with both MHC-I and ERp57, which was not present in US11 siRNA treated cells (Fig 4A). To confirm the identity of the co-immunoprecipitated protein, MRC5 cells were mock treated or infected with the HCMV deletion mutants ΔUS2-6 and ΔUS2-6/US11 and a re-immunoprecipitation was performed. After dissociation of proteins immunoprecipitated with an anti-ERp57 antibody, an anti-US11 antiserum was applied. A protein, the size of US11, was co-immunoprecipitated from the ΔUS2-6 sample, but not from the ΔUS2-6/US11 sample (Fig 4B), strongly indicating that US11 interacts with the PLC in HCMV infected cells. Therefore, our data imply, that US11 binds both unassembled MHC-I HCs and, in addition, MHC-I assembled in the PLC. Possibly, US11 binds to a structure of MHC-I that is not changed during the transition from unassembled to assembled form, e.g. in the alpha-3 domain. Alternatively, US11 interacts with MHC-I in different manners, one that can target the unassembled MHC-I for degradation and another that leads to a prolonged interaction with assembled MHC-I in the PLC.

**Fig 5:**
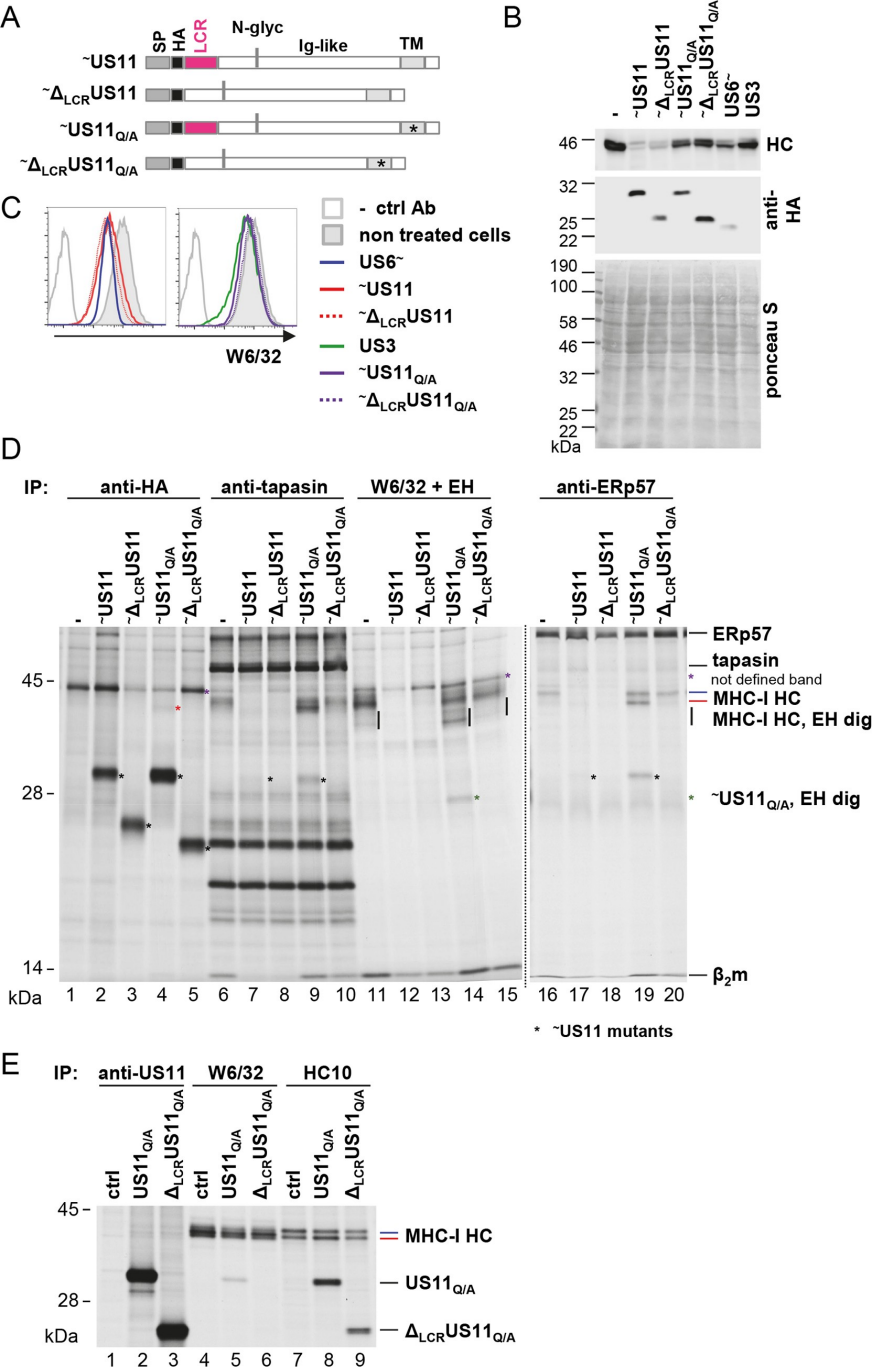
The N-terminal LCR of US11 is required for MHC-I ER retention but not for degradation. **(A)** Schematic presentation of HA-tagged US11 mutants stably transduced into HeLa cells. SP, signal peptide; HA, HA-tag, LCR, low-complexity region; N-glyc, N-glycosylation site; Ig-like, immunoglobulin-like domain; TM, transmembrane domain; asterisk, Q192A mutation. **(B)** Whole cell lysates of HeLa cells expressing HA-tagged US11 variants and US6-HA and US3 were analyzed by Western blot using HC10 for detection of MHC-I HC and anti-HA for detection of viral proteins. Equal loading of lysates was controlled by Ponceau S staining. **(C)** Analysis of MHC-I cell surface expression by flow cytometry using W6/32. **(D)** Stably transduced HeLa cells were labeled with [^35^S]-Met/Cys for 2 h and co-immunoprecipitation was performed using anti-HA, anti-tapasin, W6/32 or anti-ERp57 antibodies. W6/32 samples were subjected to EndoH (EH) digest. Black asterisk indicates US11 specific bands; the green asterisk indicates EndoH digested US11. The black vertical bar marks EndoH digested MHC-I HCs. The red asterisk marks co-immunoprecipitated endogenous HLA-A*68:02. The dotted vertical line marks separate gels. **(E)** HeLa cells stably transduced with US11 constructs as indicated were labeled with [^35^S]-Met/Cys for 2 h and immunoprecipitation was performed using anti-US11, W6/32 or HC10 antibodies. A long exposure of the gel is depicted in S9 Fig. A representative of at least two independent biological replicates with similar outcomes is shown in each panel.

In this regard we found it interesting, that US11 has a predicted low-complexity region (LCR; amino acids 28–42 [34]) N-terminal of the Ig-like domain. Since LCRs tend to be engaged in protein-protein interactions [35], we set out to investigate the importance of this domain for US11 function and interaction with MHC-I. To this end, we deleted the N-terminus (amino acids 20–44 of the full-length protein) with the LCR from US11 wild-type and from a US11 Gln192Ala (US11_Q/A_) mutant (schematic illustration in Fig 5A). The Gln192Ala mutation prevents the recruitment of Derlin-1 and subsequent MHC-I degradation, which leads to retention of MHC-I in the ER [22]. Therefore, this modification of US11 allows for interaction studies, circumventing the issue of losing substrates due to degradation. The HA-tagged US11 versions were stably transduced into HeLa cells and the cells were first checked for steady-state level expression of US11 and MHC-I. In the analysis we included HeLa cells expressing US6 and US3 to control for strong block of MHC-I peptide loading and retention, respectively. Lysates from these cells were subjected to Western blot analysis. The total level of MHC-I was strongly reduced in both US11 and Δ_LCR_US11 expressing cells, suggesting that the US11 LCR is dispensable for degradation of MHC-I (Fig 5B). The expected rescue of MHC-I expression was detected in cells expressing the US11 Q192A mutants. Flow cytometry analysis revealed that surface expression of MHC-I was downregulated both by US11 and Δ_LCR_US11 to the same extent as by US6 (Fig 5C). Expression of the US11 Q192A mutants lead to lower level of MHC-I downregulation, also when compared to US3 expressing cells, indicating that the retention is not as strong as for US3. To analyze interactions between US11, MHC-I and the PLC in more detail, we next performed co-immunoprecipitation experiments using metabolically labeled cells. (Fig 5D; immunoprecipitation using an anti-transferrin receptor control antibody is shown in S8 Fig). Whereas the LCR was dispensable for US11-dependent degradation of MHC-I molecules in the context of these stable cell lines (compare MHC-I HC levels in Fig 5D, lanes 11, 12, and 13), MHC-I ER retention caused by the Q192A mutation (compare the level of EndoH sensitive MHC-I HC in lanes 11, 14 and 15) and stabilization of MHC-I in the PLC (compare the level of MHC-I HC in lanes 6, 9 and 10 as well as in lanes 16, 19, and 20) was dependent on the LCR, as the mentioned effects was only observed by the full-lengh US11_Q/A_ mutant, but not by Δ_LCR_US11_Q/A_. This difference in function correlated with co-immunoprecipitation of the US11 mutants: US11_Q/A_ co-immunoprecipitated with W6/32 (lane 14), anti-tapasin (lane 9) and anti-ERp57 antibodies (lane 19). Most convincingly, a weak co-immunoprecipitation of wildtype US11 was observed with anti-tapasin (lane 7) and anti-ERp57 antibodies (lane 17), despite very low MHC-I level in these samples, while no co-immunoprecipitation of Δ_LCR_US11_Q/A_ was observed by any of the PLC or MHC-I reactive antibodies.

In contrast, Δ_LCR_US11_Q/A_ could be detected in association with unassembled HCs when using the mAb HC10 for immunoprecipitation, which predominantly binds to free HCs (Fig 5E, lane 9; of note, HC10 also immunoprecipitates HLA-A*68:02 HC [36, 37]). This is consistent with the finding that US11 lacking the LCR is still able to mediate degradation of MHC-I HCs. Moreover, we observed that the affinity of US11 for fully assembled MHC-I, which are recognized by the mAb W6/32, was strongly reduced (compare US11 co-immunoprecipitation in Fig 5E, lanes 5 and 8; long exposure in S9 Fig), and this was even more pronounced for the Δ_LCR_US11 mutant, demonstrating that US11 interacts with unassembled and assembled MHC-I in different manners. In conclusion, in stably transduced cells US11 interacts with unassembled MHC-I HCs and redirect them for degradation independently of the N-terminal LCR. If, however, the recruitment of ERAD is prohibited by the US11 Q192A mutation, US11 remains in a complex with assembled MHC-I molecules that are bound to the PLC and retained in the ER. This ability of US11 is dependent on the LCR, since in Δ_LCR_US11_Q/A_ expressing cells, MHC-I molecules matured as in control cells. The findings are summarized in a schematic Table in the supplementary material (S10 Fig).

**Fig 6:**
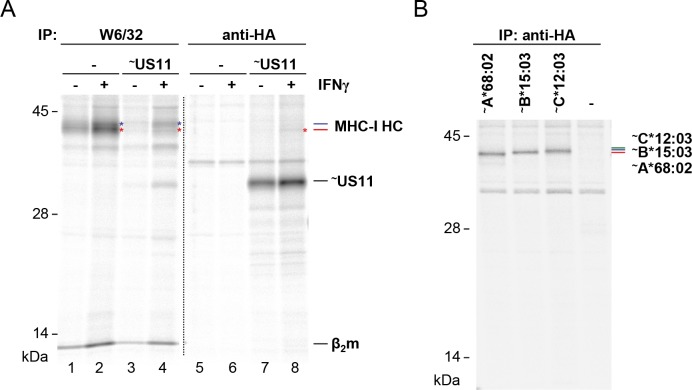
Resistant HLA-B in cells ectopically expressing US11. **(A)** Stable HA-US11-HeLa cells (~US11) or control cells (-) were treated for 36 h with IFN-γ (500 U/ml) or left untreated. Cells were labeled with [^35^S]-Met/Cys for 2 h and immunoprecipitation was performed using indicated antibodies. Distinct MHC-I HCs are indicated with blue and red asterisks. A representative of two independent biological replicates with similar outcomes is shown. **(B)** Hela cells were transiently transfected with HA-tagged MHC-I encoded by the pUC-IP vector. At 20 h post-transfection an immunoprecipitation experiment as described in (A) was performed.

An additional observation from this experiment that appeared contradictory, was the lack of resistant MHC-I molecules in cells stably expressing US11. However, in contrast to low MHC-I expression levels in these HeLa cells, MHC-I is massively induced upon HCMV infection [33]. To obtain expression levels comparable to infected cells, we treated the US11-expressing cells with IFNγ. Under these conditions MHC-I was more readily detectable even in the presence of US11 (Fig 6A, lane 4). In control HeLa cells, the lower MHC-I HC band (red asterisk) appeared stronger after IFNγ induction than the upper band (blue asterisk), whereas in US11-expressing cells the lower band was weaker than the upper one. This strongly suggests that the lower MHC-I molecule is more sensitive to degradation. To determine the identity of the MHC-I HC bands, HA-tagged versions of the single HLA-A, -B and -C molecules expressed in HeLa cells (HLA-A*68:02, B*15:03, and C*12:03) [38], were expressed by transient transfection and subsequently their SDS-PAGE separation properties were determined after immunoprecipitation (Fig 6B). The obtained pattern suggests that the lower US11 sensitive MHC-I HC corresponds to HLA-A*68:02. The upper band that was more resistant to US11 appeared as a smear and could comprise both HLA-B*15:03 and C*12:03. In conclusion, under conditions of a high US11/MHC-I ratio, US11 selectivity is less pronounced. Elevated levels of MHC-I leads to a distinct preference of US11 for degradation of HLA-A.

### The US11 LCR influences peptide loading of HLA-B*15:03

Our data shows that US11 binds to MHC-I HCs and redirects them for proteasomal degradation independently of the LCR. We asked what could be the purpose of the LCR in complex with assembled MHC-I. The PLC not only maintains empty or suboptimally loaded assembled MHC-I molecules in a peptide-receptive state, but also selects peptides with stabilizing properties [39–41]. To clarify the role of US11 in the PLC, we next investigated whether US11 can influence peptide selection. To this end, the MHC-I ligandome of HeLa cells overexpressing US11_Q/A_, Δ_LCR_US11_Q/A_, or US3 was compared to non-transduced control cells. US3 was included as a control because it retains MHC-I in the ER and was reported to interact with the PLC [10, 13, 42]. The ligandome was determined by LC-MS/MS after isolation of MHC-I by the mAb W6/32 and elution of peptides [43]. The stable cell lines were analyzed in two replicates, the results of which clustered tightly. For better binding prediction, only 9-mer peptides were used for further analysis and assigned as ligands of HLA-A*68:02 or B*15:03 if their affinities were predicted to be <500 nM by NetMHC3.4 [44]. If a ligand was classified as a binder to both MHC-I molecules, it was assigned as a ligand to the one for which a higher affinity was predicted.

**Fig 7:**
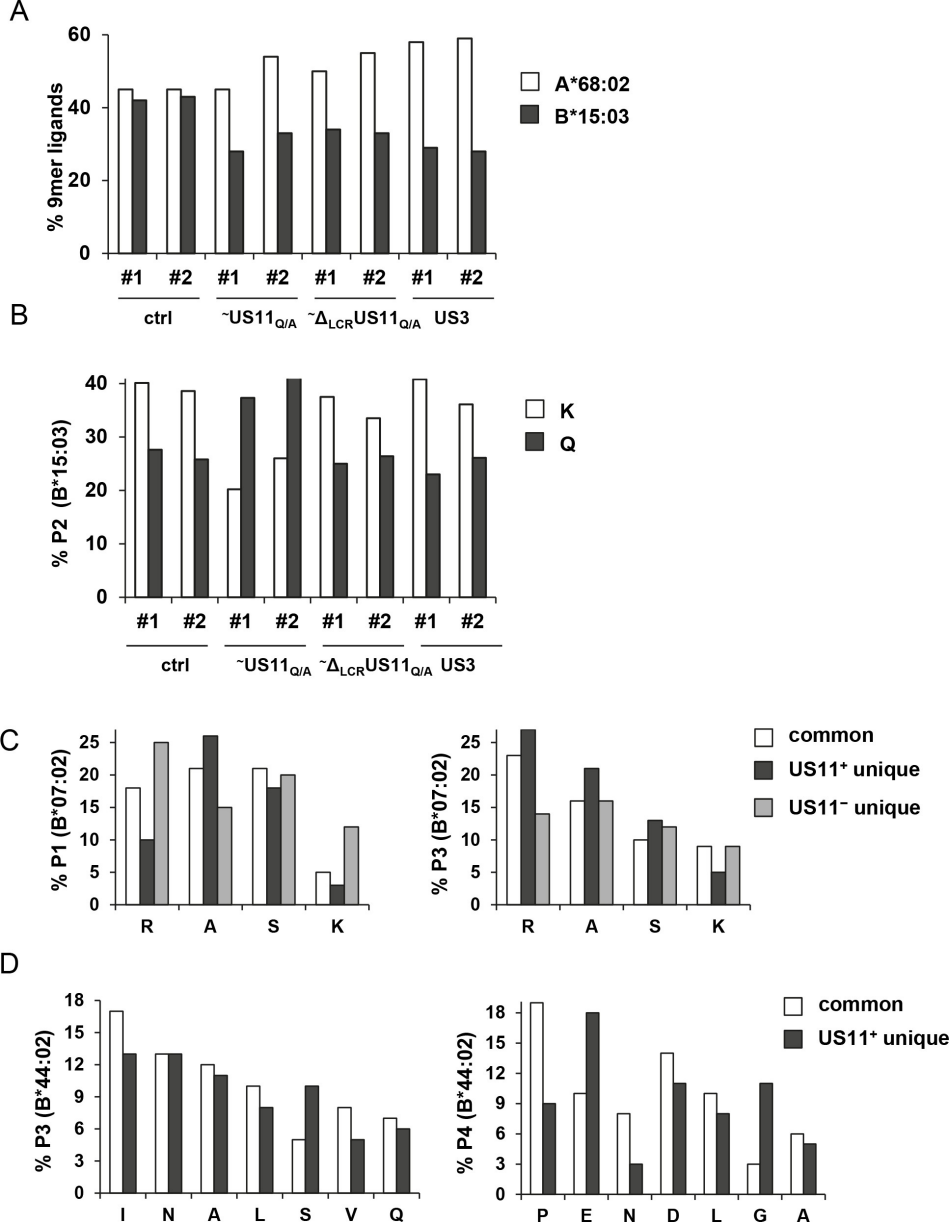
Anchor residue usage of HLA-B ligands is modified by US11. **(A-B)** Analysis of HLA class I ligands was performed in control HeLa cells or HeLa cells stably transduced with HA-tagged (~) US11_Q/A_, Δ_LCR_US11_Q/A_ or US3. Cells were collected and MHC-I molecules were isolated using the mAb W6/32. Peptide ligands were eluted and analyzed by mass spectrometry. (**A**) The relative distribution of MHC-I specific 9-mer ligands between HLA-A*68:02 and B*15:03 is shown. (**B**) The frequency of P2 peptide anchor residues of HLA-B*15:03 9-mer ligands was determined and depicted as percentage of total pool at that specific position. Two independent biological replicates of the experiment are shown (#1 and #2). **(C-D)** Pooled #1 and #2 ligands from Fig 1A predicted by NetMHC3.4 to bind to HLA-B*07:02 and B*44:02 with an affinity of <500 and <1000 nM, respectively, were divided into common and unique ΔUS2-6 and ΔUS2-6/US11 ligands respectively (S13A Fig). From these pools the frequency of specific amino acids (x-axis) at positions P1 and P3 of HLA-B*07:02 (C) and positions P3 and P4 of HLA-B*44:02 (D) was determined and depicted as percentage of total pool at that specific position.

Very similar amounts of ligands were found for HLA-A*68:02 and HLA-B*15:03 in control cells (42–45% each of all 9-mers, Table 1 and Fig 7A). In transduced cells (US11_Q/A_, Δ_LCR_US11_Q/A_, US3), however, the percentage of HLA-B*15:03 derived ligands decreased (28–33% of all 9-mers), pointing to an advantage for HLA-A*68:02 expression or loading in the transduced cells. Changes in MHC-I antigen presentation upon lentiviral transduction has been observed also by others [45]. In US11_Q/A_ expressing cells the overall amount of 9-mer ligands was strongly reduced compared to the other samples (Table 1), suggesting that US11_Q/A_ impaired proper peptide loading. Of note, this was not the case for HeLa cells expressing ~Δ_LCR_US11_Q/A_ or US3.

**Table 1:** Number of 9-mer MHC-I ligands isolated from HeLa cell lines.

HeLa	ctrl		~US11_Q/A_		~Δ_LCR_US11_Q/A_		US3	
Replicate	#1	#2	#1	#2	#1	#2	#1	#2
**9-mers_tot_**	666	869	248	680	558	860	843	1005
A*68 (*%*)	299 (*45*)	391 (*45*)	112 (*45*)	368 (*54*)	279 (*50*)	475 (*55*)	493 (*58*)	589 (*59*)
B*15 (*%*)	281 (*42*)	372 (*43*)	73 (*28*)	220 (*32*)	188 (*34*)	283 (*33*)	248 (*29*)	286 (*28*)

To gain a more detailed view of the effect of ~US11_Q/A_ on MHC-I peptide loading, the usage of ligand anchor residues was compared between HeLa cell lines. HLA-B*15:03 prefers ligands with a lysine or glutamine at position 2 (P2) and a tyrosine or phenylalanine at the C-terminal position (P9). While no change in the usage of the P9 anchor residues was observed between cells (S11 Fig, right panel), the usage frequency of lysine and glutamine at P2 was inversed in US11_Q/A_ expressing HeLa cells (Figs 7B and S11, right panel). In these cells lysine was found at P2 in 15–20% of the HLA-B*15:03 ligands and glutamine in 37–39%, whereas in wild-type HeLa and in the other transduced cell lines, including the ~Δ_LCR_US11_Q/A_ expressing cells, lysine was the most common residue and glutamine was less frequently used, 33–41% and 23–28%, respectively. We did not detect changes in the ligandome of HLA-A*68:02 (S11 Fig, left panel). These data suggest that the LCR of US11 interferes with peptide loading of distinct MHC-I molecules.

To analyze whether US11 affects the MHC-I ligandome also in HCMV infected cells, we used the data sets from Fig 1. However, no clear changes in the HLA-B*07:02 and B*44:02 ligandomes were observed when we compared the cells infected with ΔUS2-6 (US11_pos_) and ΔUS2-6/US11 (US11_neg_) (S12 Fig). These HLA-B allotypes are very strict in their usage of P2 anchor residues with a high frequency of proline at P2 in B*07:02 peptides and glutamic acid at P2 in B*44:02 peptides and might therefore be more resistant to US11-mediated peptide modification. To better visualize possible changes, the ligandomes were divided into pools of common and unique peptides in the ΔUS2-6 (US11_pos_) and ΔUS2-6/US11 (US11_neg_) samples (S13A Fig). Using this setting, we found that unique HLA-B*07:02 peptides in the ΔUS2-6 (US11_pos_) pool varied at P1, but not much at P3 (Figs 7C and S13B) compared to the common pool. Interestingly, unique HLA-B*07:02 peptides in the ΔUS2-6/US11 (US11_neg_) pool showed an opposite effect at P1, emphasizing that the effect is US11-specific. Regarding HLA-B*44:02, only three unique peptides could be defined in the ΔUS2-6/US11 (US11_neg_) pool (S13A Fig) and therefore changes in the ligandome could not be strengthened by this group of peptides. However, we observed that the commonly used proline at P4 [46] was reduced by US11 and instead usage of glutamate was increased. As only a few HLA-A peptides were detected in the ΔUS2-6 (US11_pos_) unique pool (18 and 15 for A*02:01 and A*29:02, respectively) this data set could not be applied conclusively for analysis of peptide modification. The increased frequency of glutamate at P2 in HLA-A*29:02 ligands of the ΔUS2-6 (US11_pos_) unique pool is, nonetheless, an interesting observation (S14 Fig, right panel).

## Discussion

**Fig 8:**
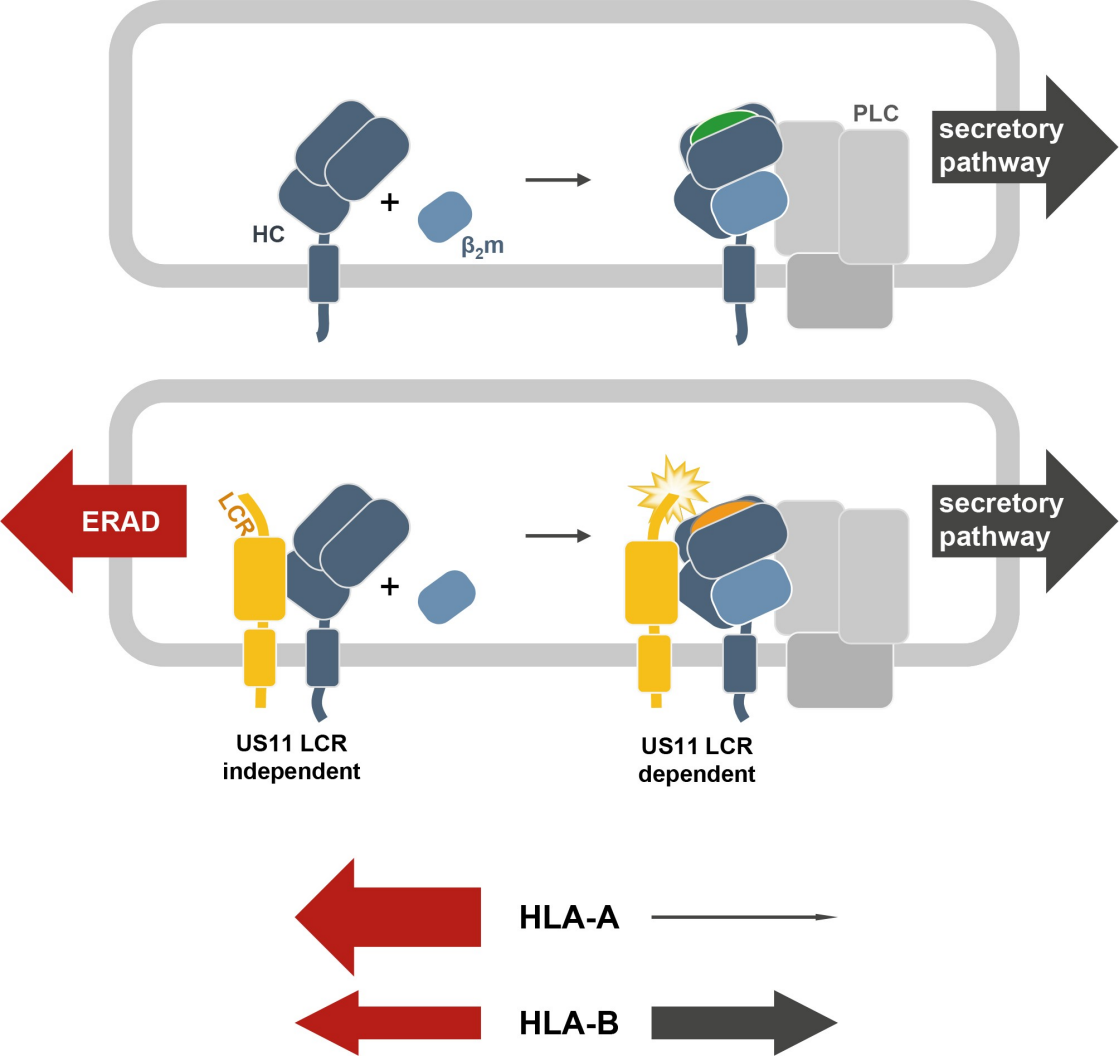
Model for US11 regulation of HLA-A and HLA-B. The HC of MHC-I dimerizes with β_2_m and is recruited to the PLC, where peptide (green) loading takes place. The stabilized MHC-I/peptide complex leaves the PLC for cell surface expression through the secretory pathway. In the presence of US11 (yellow), MHC-I molecules are redirected for proteasomal degradation via an ERAD pathway. This function can be executed by US11 also without the LCR. HLA-B HCs comprise an intrinsic resistance against US11-mediated degradation and dimerize more efficiently with β_2_m in the presence of US11. In an LCR-dependent manner US11 modifies peptide (orange) loading. The relative effect of US11 on HLA-A and HLA-B is depicted with arrows, the thickness of which is indicative of the efficacy of degradation (red) and surface expression (black).

The human cytomegalovirus is the largest of all human herpesviruses, both with respect to its genome length and numbers of transcribed ORFs [47]. Whereas all herpesviruses interfere with the host immune defense at some level, the unique infection biology of cytomegaloviruses has come along with the evolution of a large array of highly specialized immune evasive genes and reprogramming of multiple immune functions without compromising the human host [48]. HCMV controls distinct checkpoints of the MHC-I antigen presentation pathway by at least five gene products [6, 49] and most likely even more genes are involved in a less defined manner [33, 50–52]. The human immune system can cover the presentation of a wide breadth of pathogen derived antigens owing to the three extraordinary polymorphic MHC-I genes each individual possesses. This unique and continuing diversification of human HLA may have promoted the emergence of multiple HCMV genes blocking MHC-I antigen presentation. Obviously, it would be less strategic to attack the MHC-I pathway only at a checkpoint shared by all HLA gene products since they display differential immunological impacts. Nevertheless, so far, specificities for representative alloforms of HCMV encoded inhibitors have not been comprehensively studied. Here, we show that a prime target for US11-mediated degradation is HLA-A locus products, whereas HLA-B resists this effect. Instead, US11 has acquired an independent function in its N-terminal LCR to manipulate peptide loading of HLA-B molecules (model in Fig 8).

Advancements in mass spectrometry analysis [53] encouraged us to analyze the MHC-I ligandome of HCMV-infected MRC-5 fibroblasts. The primary aim was to identify novel CD8+ T-cell restricted epitopes (Lübke *et al*., manuscript submitted). During these studies we came across a remarkable phenomenon regarding US11: whereas the quantity of HLA-A allotype (HLA-A*02:01, A*29:02)-derived peptides was reduced by US11 as expected, this was not the case for the HLA-B (HLA-B*07:02, B*44:02) and -C (HLA-C*05:01, C*07:02) ligandomes. This effect was even more pronounced when only HCMV derived MHC-I ligands were considered. Flow cytometry measurements of infected fibroblast and epithelial cells demonstrated that US11 slightly reduced the level of HLA-B*07:02 surface expression, however, this was much less pronounced as compared with HLA-A*02:01. Altogether, this indicates that in HCMV-infected cells US11 is not able to degrade HLA-B efficiently. The observed higher US11-resistance by HLA-B is in agreement with recent observations of HCMV-infected cells [54, 55] and plasma membrane profiling studies of THP-1 cells expressing US11 [19].

### HLA-B molecules dimerize with β_2_m and mature in the presence of US11

To assess the effect of US11 on a larger panel of various MHC-I molecules, we elaborated a convenient and fast flow cytometry based assay system measuring MHC-I cell surface disposition after transient transfection of HeLa cells (Fig 2A and 2B). MHC-I molecules were designed to express an inert HA-epitope tag at the N-terminus to overcome the need for allotype-specific antibodies. In this way, we measured unrestricted expression of HLA-B allotypes on the cell surface, whereas HLA-A allotypes were strongly reduced.

Unexpectedly, we observed in pulse-chase experiments that US11 substantially affected HLA-B expression in the early secretory pathway (Fig 4C), despite the unchanged density on the cell surface. However, at variance with HLA-A locus products, HLA-B alloforms were able to dimerize with β_2_m and mature in the presence of US11. HLA-B molecules thus escaped US11 and accumulated on the surface to the same extent as in US11-negative cells. The functional expression of HLA-B*07:02 in the presence of US11 could be further demonstrated by binding to LIR1.

Furthermore, we found that the level of polymorphic MHC-I synthesized in the ER strongly affects the efficacy and selectivity of US11-mediated degradation. In HCMV-infected cells transcription and biosynthesis of MHC-I is highly upregulated [33]. This could explain why US11 does not reach the critical level required to degrade HLA-B, while HLA-A is still efficiently recruited to ERAD, as we observed also in IFNγ-induced HeLa cells stably expressing US11. This underlines the relevance of the strict regulation of US11 expresssion via the HRD1-dependent autoregulatory loop [24, 25]; in the absence of MHC-I substrates US11 itself is targeted to ERAD degradation, controlled by HRD1.

We have begun to address the molecular basis of HLA-B resistance. The shorter cytosolic tail of HLA-B alleles confers some level of resistance, as described previously for US11 [31] and also for HIV-1 encoded Nef [56], but was not a pivotal factor for US11 in our experimental setup. The results obtained with β_2_m-deficient FO-1 cells showed that β_2_m is not critically involved in resistance against degradation. Indeed, this demonstrated that this resistance should be an intrinsic property of HLA-B HCs, which is now an object of further investigation.

### A molecular model for US11 LCR interaction and function

US11 possesses an LCR sequence between its signal peptide and the Ig-like luminal domain. This region is 15 amino acids long (residues 28–42) and contains seven proline residues. Such structurally undefined regions often function as multiprotein interaction hubs, e.g. found in chaperons [35]. Furthermore, LCRs may have advantages for faster adaptation and evolution [57]. Our analysis revealed that the LCR of US11 is required for several features of US11 that are possibly interconnected. Firstly, the ability of US11 to interact with folded heterodimers of MHC-I HC and β_2_m, as defined by recognition by the mAb W6/32 [58], is dependent on the LCR. Secondly, we found that US11 interacts with the PLC most likely via binding to MHC-I, since low MHC-I expression levels resulted in low US11 co-immunoprecipitation with the PLC. Again, this interaction was dependent on the US11 LCR, confirming that only folded heterodimeric MHC-I molecules interact with the PLC [5, 59]. US11 with a Q192A mutation is not able to forward MHC-I to the ERAD pathway. As a consequence MHC-I is retained in the ER [15, 22]. This requires a stable interaction between US11 and assembled MHC-I heterodimers that involves the LCR of US11, because deletion of the LCR rescued MHC-I transport through the secretory pathway in the context of the US11_Q/A_ mutant. However, the ability of US11 to forward MHC-I HC for ERAD degradation is not affected by the deletion of the LCR. In cells stably expressing Δ_LCR_US11 a strong reduction of MHC-I was observed, in accordance with the finding that Δ_LCR_US11_Q/A_ is still able to stably interact with unassembled MHC-I HCs. This indicates that US11 can initiate retrotranslocation and degradation of MHC-I without its LCR at a stage before heterodimeric MHC-I molecules assemble. The targeting of unassembled MHC-I in β_2_m deficient cells has been observed previously [60].

The most remarkable feature of the US11 LCR, however, was its ability to manipulate HLA-B*15:03 peptide ligands. The usage of the N-terminal P2 position of the ligand anchor residue was changed in a way that the frequently appearing lysine was strongly reduced and the generally less used glutamine emerged most frequently in the presence of US11. Control cells, Δ_LCR_US11_Q/A_ cells or cells expressing US3, which also binds to and retains MHC-I heterodimers, did not exhibit this effect on HLA-B*15:03, indicating that it is a specific feature of the US11 LCR that interferes with peptide loading. We observed this change only for the N-terminal anchor residues and not for the C-terminal. We were not able to define any similar changes in the ligandome of HLA-A*68:02.

The HLA-B molecules HLA-B*07:02 and B*44:02 present in MRC-5 fibroblast do not allow for measurable modifications at P2, since the P2 residue is strongly fixed for these molecules (by proline and glutamate, respectively). However, unique HLA-B*07:02 and HLA-B*44:02 ligands in MRC-5 cells infected with the ΔUS2-6 HCMV mutant virus expressing US11, displayed changes in the neighboring P1 and P4, respectively, suggesting that US11 influences the peptide selection for a broad range of HLA-B allotypes.

The recently resolved structure of the PLC [5] provides a molecular basis to model manipulation of HLA-B by US11. The MHC-I peptide binding groove is deeply buried in the PLC with the F-pocked that binds the C-terminal peptide anchor residue pointing inwards into the center of the PLC. The opposite side of the MHC-I HC is the only region of MHC-I still accessible for further protein interactions. This interface is also used by HCMV encoded US2 and adenovirus encoded E3-19K as demonstrated in resolved crystal structures [14, 61]. If US11 also binds to this particular surface, which is likely, since US11 interacts with MHC-I during its processing through the PLC, the LCR could be in the vicinity of MHC-I residues contributing to the formation of pockets that fix the N-terminal part of the peptide.

Whereas HLA-B allotypes are at the forefront in studies determining protective and sensitizing MHC-I in HIV and HCV infections [62, 63], such observations have not been made for HCMV or other herpes viruses. This goes well together with the suggestion that HLA-A is more important to control co-evolving DNA viruses [64]. The differential targeting of HLA-A and -B by US11 underlines this view and implies that complete block of antigen presentation by HLA-A is crucial for the virus to cope with highly specific CD8+ T-cells. Unlike HLA-A, a large fraction of HLA-B allotypes contains the Bw4 motif recognized by inhibitory KIRs (Killer cell immunoglobulin-like receptors) on NK cells [65]. Therefore, the costs for allowing a reduced level of HLA-B surface expression, yet, with a modified peptide repertoire, might be tolerated by HCMV, in order to dampen NK cell activation.

Future studies will provide more insight into the mechanism how the US11 LCR alters the quality of MHC-I peptide ligands and the functional ramifications of this alteration. It is conceivable that this feature of US11 could confound CD8+ T-cell recognition of HCMV infected target cells. The initial priming of CD8+ T precursors is believed to occur via cross-presentation [66, 67], i.e. by non-infected dendritic cells in the absence of US11. Thus, the quality of the MHC-I presented peptides might differ significantly between productively HCMV-infected cells, in which US11 is actively expressed and professional APC priming the CD8+ T-cells. Whether US11 will impact the formation of memory cells and memory inflation is not predictable. However, it will be of great interest to learn whether Rh189 (RhCMV US11 homolog)-induced non-canonical CD8+ T-cell restricted epitopes [29] are also dependent on the N-terminal part of the protein and could be a result of manipulation of peptide loading. Although an LCR is not predicted in the N-terminus of Rh189, it still contains some conserved residues (S15 Fig), possibly important for interaction with assembled MHC-I.

## Materials and methods

### Cells and transfection of plasmids and siRNA

MRC-5 fibroblasts (ECACC 05090501; HLA-A*02:01, A*29:02, B*07:02, B*44:02, C*05:01, C*07:02), HeLa (ATCC CCL-2; HLA-A*68:02, B*15:03, C*12:03; ATCC CCL-2), and US6-HA-HeLa cells [68], ARPE-19 (ATCC CRL-2302), the melanoma cell line FO-1 [69] and HEK293T (ATCC CRL‐11268) cells were grown in DMEM supplemented with 10% FCS, penicillin and streptomycin. HeLa cells were tranfected with Superfect (Qiagen) and FO-1 cells with Jetprime (Polyplus Transfection). Small interfering RNA (siRNA) targeting US11 (ACACUUGAAUCACUGCCACCCCC) was purchased from Riboxx. Knock-down experiments were performed using Lipofectamin RNAiMax Reagent (Invitrogen).

### Viruses

The recombinant HCMV mutants ΔUS2-6/US11 and ΔUS2-US11 was generated according to a previously published procedure [70] using the BAC-cloned AD169varL genome pAD169 [71] as parental BAC. Briefly, PCR fragments was generated using the primer pair KL-DeltaUS11-Kana1 CAAAAAGTCTGGTGAGTCGTTTCCGAGCGACTCGAGATGCACTCCGCTTCAGTCTATATACCAGTGAATTCGAGCTCGGTAC and KL-DeltaUS11-Kana2 TAAGACAGCCTTACAGCTTTTGAGTCTAGACAGGGTAACAGCCTTCCCTTGTAAGACAGAGACCATGATTACGCCAAGCTCC for the ΔUS2-6/US11 mutant and the primer pair KL-DeltaUS7-Kana1 ACCTTTTGTGCATACGGTTTATATATGACCATCCACGCTTATAACGAACCTAACAGTTTACCAGTGAATTCGAGCTCGGTAC and KL-DeltaUS11-Kana2 TAAGACAGCCTTACAGCTTTTGAGTCTAGACAGGGTAACAGCCTTCCCTTGTAAGACAGAGACCATGATTACGCCAAGCTCC for the ΔUS2-US11 mutant and the plasmid pSLFRTKn [72] as template DNA. The PCR fragment containing a kanamycin resistance gene was inserted into the parental BAC by homologous recombination in *E*. *coli*. Correct mutagenesis was confirmed by Southern blot and PCR analysis. Recombinant HCMVs including TB40/E ΔUS2-6 [73] were reconstituted from HCMV BAC DNA by Superfect (Qiagen) transfection into permissive MRC-5 fibroblasts. Virus titers were determined by standard plaque assay.

Production of lentiviruses was performed as described previously [38]. At 48 h post transfection the supernatant was collected and filtered through a 45 μm filter prior to transduction of HeLa cells by centrifugal enhancement. When selected, the cells were cultivated in normal medium for 3–4 days before treatment with 5 μg/ml puromycin (Sigma).

### Antibodies

The following antibodies were applied: W6/32 (anti-pan-HLA-A,B,C assembled with β_2_m and peptide, [58]), BB7.2 (anti-HLA-A2 [74]), BB7.1 (anti-HLA-B7 [74]), TT4-A20 (anti-HLA-B44 [75]), HC10 and HCA2 recognizing free HLA-B/C and HLA-A heavy chains, respectively [76], anti-CD71 (immunotech), anti-CD85j (LIR1; Miltenyi) mouse and rabbit anti-HA antibodies (Sigma), anti-ERp57 (Millipore), APC-coupled anti-mouse antibodies (BD Pharmingen). Polyclonal anti-tapasin and anti-US11 anti-sera were raised by immunization of rabbits (Genscript) with synthetic peptides (aa 418–428 and 90–103, respectively).

### Molecular cloning

The tapasin signal peptide sequence was amplified in front of a human influenza hemagglutinin (HA)–tag and cloned into *XhoI* and *PstI* of pIRES-EGFP (Tpn-SP-pIRES-EGFP; CMV IE promoter). HLA-A*02:01, HLA-B*07:02, HLA-C*07:02, CD99 were amplified from cDNA prepared from MRC-5 cells and HLA-A*68:02 and HLA-B*15:03 from cDNA from HeLa cells. HLA-B*44:02 and HLA-B*44:05 were described previously [38]. The cDNA clones for HLA-A*01:01 (NM_001242758, BC003069), HLA-A*03:01 (NM_002116) and HLA-B*08:01 (AK292226, BC091497) have been purchased from Source Bioscience, Nottingham, UK. HLA-A*29:02 (IMGT/HLA database) was synthesized as a gBlock (Integrated DNA Technologies, Inc.) gene fragment. Irrespective of their source, all MHC-I sequences were used as a template for further amplification using specific primers (Table 2). PCR products were digested with *PstI* or *NsiI* and *BamHI* restriction enzymes and cloned into Tpn-SP-pIRES-EGFP. Sequenced inserts were subsequently subcloned into the puc2CL6IP (pUC-IP, with spleen focus-forming virus U3 promoter) lentiviral vector [38] using the restriction sites *NheI* and *BamHI*. HA-HLA-C*12:03 was purchased in pcDNA3.1 from Biocat and subcloned into puc2CL6IP. US11 and US3 cDNA was amplified from AD169 HCMV DNA and cloned into pIRES-EGFP via *NheI* and *BamH* or into puc2CL6IP and puc2CL6-IRES-*EGFP* (pUC-*EGFP*) lentiviral vector using the same enzymes. Point mutation in US11 was inserted using the QuickChange II XL Site-Directed Mutagenesis Kit (Agilent) following the protocol described by the manufacturer.

**Table 2:** Primer sequences used for cloning.

Construct	Primer
Tapasin signal peptide + HA	1. CGTCTCGAGATGAAGTCCCTGTCTCTGCTCC 2. CCTACTGCAGCTGCGTAATCTGGAACATCGTATGGGTACACCGCGGGTCCTGCTGA
HLA-A*02:01	1. CGAGCTAGCATGGCCGTCATGGCGCCC 2. CGAGGATCCTCACACTTTACAAGCTGTGAGAG
HA-A*02:01	1. CGTATGCATTAGGAGGCTCTCACTCCATGAGG 2. CGAGGATCCTCACACTTTACAAGCTGTGAGAG
HA-A*68:02	1. CGTATGCATTAGGAGGCTCCCACTCCATGAGG 2. CGAGGATCCTCACACTTTACAAGCTGTGAGAG
HA-A*29:02	1. CGTATGCATTAGGAGGCTCTCACTCCATGAGGTATTTCTTC 2. CGAGGATCCTCACACTTTACAAGCTGTGAGAGACATATC
HA-A*01:01	1. CGTATGCATTAGGAGGCTCCCACTCCATGAGG 2. GCAGGATCCTCACACTTTACAAGCTGTGAGAGAC
HA-A*03:01	1. CGTATGCATTAGGAGGCTCCCACTCCATGAGG 2. GCAGGATCCTCACACTTTACAAGCTGTGAGGG
HA-B*07:02	1. CGTCTGCAGTAGGAGGCTCCCACTCCATGAGG 2. CGAGGATCCTCAAGCTGTGAGAGACACATCAG
HA-B*44:02	1. CGTATGCATTAGGAGGCTCCCACTCCATGAGG 2. CGAGGATCCTTCAAGCTGTGAGAGACACATC
HA-B*44:05	1. CGTATGCATTAGGAGGCTCCCACTCCATGAGG 2. CGAGGATCCTTCAAGCTGTGAGAGACACATC
HA-B*15:03	1. CGTATGCATTAGGAGGCTCCCACTCCATGAGG 2. CGAGGATCCTCAAGCTGTGAGAGACACATCAG
HA-B*08:01	1. CGTCTGCAGTAGGAGGCTCCCACTCCATGAGG 2. CGAGGATCCTCAAGCTGTGAGAGACACATCAG
HA-C*07:02	1. CGTATGCATTAGGATGCTCCCACTCCATGAGG 2. CGAGGATCCTCAGGCTTTACAAGTGATGAGAG
HA-CD99	1. GGACTGCAGTAGGAGCCCCGGATGGTGGTTTC 2. GCAGGATCCTATTTCTCTAAAAGAGTACGCTGAAC
HA-A3ΔCKV	1. CGAGCTAGCGCTACCGGACTCA 2. CGAGGATCCTCAAGCTGTGAGGGACACATCAGA
HA-B7+CKV	1. CGAGCTAGCGCTACCGGACTCA 2. CGAGGATCCTCACACTTTACAAGCTGTGAGAGACACATCAGAGC
HA-US11	1. GCTCTGCAGTAGGAGGAATGCCTGAATTATCCTTGACTCTT 2. GGAGGATCCTCACCACTGGTCCGAAAAC
HA-Δ_LCR_-US11	1. GCTCTGCAGTAGGAGGAGTTTCGGAGTACCGAGTAGAGTATTC 2. GGAGGATCCTCACCACTGGTCCGAAAAC
US11	1. CGAGCTAGCGCCGCCACCATGAATCTTGTAATGCTTATTCTAGCC 2. CGAGGATCCTCACCACTGGTCCGAAAAC
Δ_LCR_-US11	1. CGAGCTAGCGCCGCCACCATGAATCTTGTAATGCTTATTCTAGCC 2. CGAGGATCCTCACCACTGGTCCGAAAAC
US3	1. CGTGCTAGCATGAAGCCGGTGTTGGTGCT 2. GCAGGATCCTTAAATAAATCGCAGACGGGCGC
US10	1. CGTGCTAGCATGCTACGCCGGGGAAGC 2. GCAGGATCCTTATTCGCGAGGTGGATAATAACCGC

### MHC-I ligandome analysis

MHC-I ligands were isolated by standard immunoaffinity purification using the mAb W6/32 as described previously [43]. LC-MS/MS analysis of MHC-I ligand extracts using nanoflow HPLC (RSLCnano, Thermo Fisher) on a 50 μm × 25 cm PepMap RSLC column (Thermo Fisher) with a gradient ranging from 2.4 to 32.0% acetonitrile over the course of 90 min. Eluted peptides were analyzed in an online-coupled LTQ Orbitrap XL mass spectrometer (Thermo Fisher) using a top 5 CID (collision-induced dissociation) method. The procedure for label-free quantification (LFQ) of relative HLA ligand abundances was performed as follows: total injected peptide amounts of paired samples were normalized and LC-MS/MS analysis was performed in five technical replicates for each sample. For normalization, the relative amounts of substance in paired samples were determined by calculating the summed area of peptide identifications in dose-finding LC-MS/MS runs and the samples were adjusted accordingly by dilution. Relative quantification of HLA ligands was performed by calculating the area under the curve of the corresponding precursor extracted ion chromatograms (XIC) using ProteomeDiscoverer 1.4 (Thermo Fisher). For Volcano plots, the ratios of the mean areas of the individual peptides in the five LFQ-MS runs of each sample were calculated and two-tailed t-tests implementing Benjamini-Hochberg correction were performed using an in-house R script (v3.2). Data processing and spectral annotation was performed as described previously [77]. In brief, the Mascot search engine (Mascot 2.2.04; Matrix Science, London, UK) was used to search the human proteome as comprised in the Swiss-Prot database (20,279 reviewed protein sequences, September 27th 2013) without enzymatic restriction. Oxidized methionine was allowed as a dynamic modification. The false discovery rate was estimated using the Percolator algorithm [78] and set to 5%. Peptide lengths were limited to 8–12 amino acids for HLA class I. Protein inference was disabled, allowing for multiple protein annotations of peptides. HLA annotation was performed using NetMHC [44] (v3.4), annotating peptides with IC50 scores below 500 nM as ligands of the corresponding HLA allotype. In cases of multiple possible annotations, the HLA allotype yielding the lowest IC50 score was selected.

### Flow cytometry analysis

MRC-5 and ARPE-19 cells were detached with Accutase (Sigma), treated with FcR blocking reagent as recommended by manufacturer (Miltenyi) and stained with antibodies diluted in 3% FCS/PBS. Cells were washed in 3% FCS/PBS supplemented with DAPI and fixed in 4% paraformaldehyde. Cells were analyzed by FACS Canto II (Becton Dickinson).

For analysis of MHC-I expression after transient transfection of HeLa cells US11 variants or a control pIRES-EGFP plasmid together with HA-tagged HLA-alleles or control molecules in puc2CL6IP were co-transfected using SuperFect (Qiagen). At 20 h post-transfection, cells were detached with trypsin and measured as described above. LIR-1 binding was analyzed by incubating the cells with recombinant protein [79] and subsequently incubating the cells with an APC-coupled anti-CD85j (LIR-1) antibody.

Acquired data was analyzed by FlowJo (v10.1, Tree Star Inc.). For statistical analyses Mann-Whitney U-test or one-way ANOVA followed by Tukey’s or Dunnett´s multiple comparison test were performed using the GraphPad Prism 6 Software. A p-value <0.05 was considered significant (*, p<0,05; **, p<0,005; ***, p<0,0005).

### Immunoprecipitation

Immunoprecipitation was performed as described previously [38]. Briefly, cells grown in 6-well plates were washed with PBS and metabolically labeled (Easytag Express [^35^S]-Met/Cys protein labeling, Perkin Elmer) with 100 Ci/ml for various times. Cells were lysed in digitonin lysis buffer (140 mM NaCl, 20 mM Tris [pH 7.6], 5 mM MgCl2, and 1% digitonin (Calbiochem)) and cleared from membrane debris at 13,000 rpm for 30 min at 4°C. For analysis of lysates with several antibodies, identical lysates were pooled then split up into equal aliquots. Lysates were incubated with antibodies for 2 h at 4°C in an overhead tumbler before immune complexes were retrieved by protein A- or G-sepharose (GE Healthcare). Sepharose pellets were washed four times with increasing NaCl concentrations (0.15 to 0.5 M in lysis buffer containing 0.2% detergent). For a re-immunoprecipitation the washed beads were subsequently incubated with a lysisbuffer supplemented with 1% Igepal (Sigma) and 1% SDS at 95° C for five minutes. The lysisbuffer was diluted to reach a final concentration of 0.1% SDS and a subsequent immunoprecipitation was performed. Endoglycosidase H (New England Biolabs) treatment was performed as recommended by the manufacturer. Prior to loading onto a SDS-PAGE iImmune complexes were dissociated at 95°C for 5 min in a DTT (40 mM) containing sample buffer. Fixed and dried gels were exposed overnight to a phosphor screen, scanned by Typhoon FLA 7000 (GE Healthcare). For better visualization of the results contrast and light were adjusted. Where mentioned in the figure legend a short or long exposure to x-ray film was used for autoradiography.

### Western blotting

For Western blot analysis, equal amount of cells were washed in PBS and lysed in lysis buffer (50 mM Tris-HCl, pH 7.5, 150 mM NaCl, 1% Igepal, and Complete protease inhibitor (Roche)). The proteins were separated by SDS-PAGE and transferred to nitrocellulose filter. After incubation with primary antibody a peroxidase coupled secondary antibody was used and chemiluminescence was detected using a LI-COR Blot Scanner.

### RNA extraction, reverse transcription, and RT-qPCR

Mock treated or infected MRC5 cells were grown in technical replicates in a 6-well format. Cells were lysed at 24 h post-infection and total RNA was extracted using NucleoSpin kit RNA II (Macherey-Nagel) and 600ng was reverse transcribed using a QuantiTect reverse transcription kit (Qiagen) and further used for both semi-quantitative and quantitative RT-PCRs. Quantitative RT-PCRs were performed with a QuantiTect SYBR green PCR kit (Qiagen). CT values were normalized to actin (ΔCT) and plotted relative to the ΔCT values of the mock treated control cells. For the semi-quantitave analysis the following primer pairs were used: US11-ctrl3’ tggtccgaaaacatccaggg and US11-ctrl5’ ttcgatgaacctccgccctt; US10-ctrl’3 aaccgcatatcaggaggaggga and US10-ctrl’5 tcacgtgcggctgtgttattca, UL40-1 gcagctagcgccgccaccatgaacaaat and UL40-2 cgaggatcctcaagcctttttcaaggcg. For the qRT-PCR we used the primers: qUS10-1 acgacggggaaaatcacgaa and qUS10-2 cagagtagtttcggggtcgg; actin beta primers (Qiagen, Hs_ACTB_1_SG QuantiTect Primer).

## Supporting information

S1 Fig **(A)** Schematic presentation of the BAC cassette inserted in front of the genes US7-*US12* in the AD169VarL BAC mutant. **(B)** MRC5 cells were mock treated or infected with ΔUS2-6 or ΔUS2-6/US11 HCMV mutants at an MOI of 5. At 24 h post-infection, RNA was isolated and RT-PCR analysis of HCMV encoded UL40, US11 and US10 was performed. **(C)** Quantitative RT-PCR analysis of mRNA isolated in B was performed and the ΔΔCT value was determined. Statistical analysis was performed applying a Mann-Whitney U-test. **(D)** Control HeLa cells (-), US10-expressing HeLa cells, and, in addition, mock treated or infected (24 h, ΔUS2-6, ΔUS2-6/US11, ΔUS2-11; MOI 5) MRC5 cells were metabolically labeled with [^35^S]-Met/Cys for 2 h and an immunoprecipitation using W6/32 was performed. **(E)** ARPE19 cells were mock treated or infected with TB40 derived ΔUS2-6 mutant. At 48 h post-infection cells were analysed by flow cytometry as indicated. Lower panel shows the mean regulation of HLA-A*02 and HLA-B*07 by the HCMV mutant compared to mock treated cells with error bars from three independent experiments. Similarly, HLA-A*02 and HLA-B*07 regulation in MRC5 fibroblast by the AD169VarL derived ΔUS2-6 mutant compared to mock treated MRC5 cells is shown (data from experiments also shown in Fig 1D).
(TIF)

S2 Fig **(A)** The reproducibility of HLA peptidome analysis is depicted by volcano plots of HLA-I peptide abundances in biological replicates of MRC-5 cells infected with ΔUS2-6 or ΔUS2-6/US11 HCMV mutants shown in Fig 1A and 1B. **(B)** Depiction of viral peptides (given as numbers on the x-axis) identified in the ligandome analysis from Fig 1A and 1B. The y-axis shows the mean PSM values from two biological replicates. For HLA-A*02:01 and A*29:02 the eluted peptides are ordered according to their abundance in ΔUS2-6 infected cells and for B*07:02 and B*44:02 according to their abundance in ΔUS2-6/US11 infected cells.
(TIF)

S3 Fig **(A)** Uncropped gel of results shown in Fig 2C**. (B)** Gel from A with increased contrast to visualize weak bands. Blue bars indicate a band to the left with the size of US11.
(TIF)

S4 Fig HeLa cells were transiently co-transfected with US11 or a control pIRES-EGFP plasmid (CMV major IE promoter) together with the indicated HA-tagged (~) HLA molecules expressed from the pUC-IP vector (SFFV U3 promoter). At 20 h post-transfection cells were labeled with [^35^S]-Met/Cys for 15 min and chased for 0, 15 and 30 min and an immunoprecipitation experiment was performed using anti-HA antibodies. The lower panel shows a pulse-chase experiment performed in parallel using anti-TfR mAbs.
(TIF)

S5 Fig Uncropped gel shown in Fig 3A. (TIF)

S6 Fig Uncropped gel shown in Fig 3E. (TIF)

S7 Fig Efficiency of four different siRNAs directed against US11 was tested in HeLa cells stably expressing HA-tagged US11. **(A)** Western Blot analysis was performed using rabbit anti-HA antibodies, mAb HC10 and as a loading control anti-calreticulin antibodies. Cells were treated with control siRNA (c) or siRNA against US11 (1–4). Control cells without US11 expression and siRNA treatment was included in the analysis (-). US11_1 siRNA was chosen for further experiments. The sequences for the siRNA are: 1, ACACUUGAAUCACUGCCACCCCC; 2, UUGAAUCACUGCCACCAUCCCCC; 3, UCUACAUAAUAAGUUUGGCCCCC; 4, UCGCACUCUACAUAAUAAGCCCCC. **(B)** Gel shown in Fig 4B, here depicted with same contrast and light settings for all parts.
(TIF)

S8 Fig Stably transduced HeLa cells with US11 variants as indicated, were labeled with [^35^S]-Met/Cys for 2 h and co-immunoprecipitation was performed using antibodies as indicated. Two different contrast and light setting are shown (upper and lower panel).
(TIF)

S9 Fig Longer exposure of gel shown in Fig 5E.
(TIF)

S10 Fig The schematic table depicts effects of the US11 LCR sequence. The table summarizes the findings from the co-immunoprecipitation experiments shown in Fig 5. White cells indicate functions that were not analyzed in detail. In addition, in the last column, also the ability to modify MHC-I peptide loading (results shown in Fig 7) is included.
(TIF)

S11 Fig Frequency of MHC-I ligand residues determined from HeLa cells. Common HLA-A68:02 and B15:03 9-mer ligands of the biological replicates #1 and #2 (from samples described in Fig 7) are depicted as sequence logos [80]. The numbers below the logos indicate the amino acid position of MHC-I peptide ligands, with HLA-A*68:02 peptide ligands in the left panel and B*15:03 ligands in the right panel.
(TIF)

S12 Fig Frequency of MHC-I ligand residues determined from HCMV infected cells. The figure shows sequence logos [80] of the total pool of HLA-B*07:02 and B*44:02 9-mer ligands derived from replicate #1 and #2 depicted in Fig 1. The peptides were considered to be specific ligands if NetMHC3.4 [44] predicted an affinity of <500 and <1000 nM, for HLA-B*07:02 and B*44:02, respectively. The numbers below the logos indicate the amino acid position of MHC-I peptide ligands.
(TIF)

S13 Fig Frequency of HLA-B ligand residues determined from HCMV infected cells. (**A**) The figure shows the number of common and unique HLA-B ligands from cells infected with ΔUS2-6 and ΔUS2-6/US11 as Venn diagrams [81] (pooled peptides from replicates #1 and #2 as described in S12 Fig). (**B**) Groups of peptides (common and unique) shown in (A) are depicted as sequence logos [80]. The numbers below the logos indicate the amino acid position of HLA-B peptide ligands.
(TIF)

S14 Fig Frequency of HLA-A ligand residues determined from HCMV infected cells. The figure shows common and unique ligands from cells infected with ΔUS2-6 and ΔUS2-6/US11 (pooled peptides from replicates #1 and #2) as sequence logos [80]. The peptides were considered to be specific ligands if NetMHC3.4 [44] predicted an affinity of <500. The number of peptides for each group is given to the left. The numbers below the logos indicate the amino acid position of MHC-I peptide ligands.
(TIF)

S15 Fig Alignment of HCMV and RhCMV US11 orthologs. The protein sequence of US11 orthologs is shown. The predicted sequences for the signal peptide is highlighted in red, for the transmembrane segment in blue and the LCR of HCMV encoded US11 in yellow. The N-terminal region deleted in the Δ_LCR_US11 mutant is marked with a black box. Highly conserved residues are marked in black, similar residues in grey and non-conserved residues in white. Glutamine 192 of HCMV US11 is marked with a red asterisk.
(TIF)
