# Supplementary figures and images for: HLA-B locus products resist degradation by the human cytomegalovirus immunoevasin US11
Source: PLoS Pathog. 2019 Sep 17;15(9):e1008040. doi: 10.1371/journal.ppat.1008040 (PMC6764698; doi:10.1371/journal.ppat.1008040)

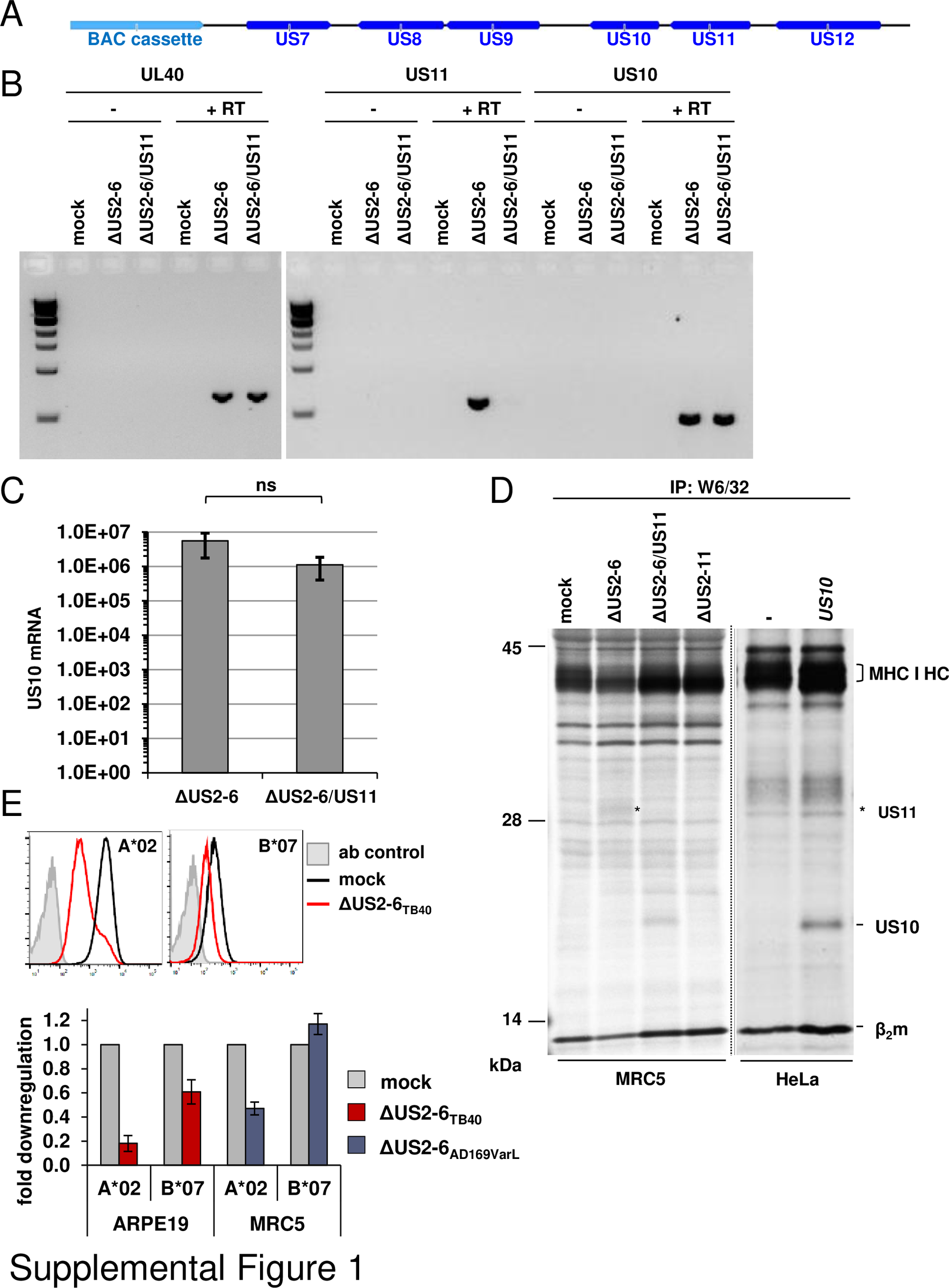

Supplement: S1 Fig — (A) Schematic presentation of the BAC cassette inserted in front of the genes US7-US12 in the AD169VarL BAC mutant. (B) MRC5 cells were mock treated or infected with ΔUS2-6 or ΔUS2-6/US11 HCMV mutants at an MOI of 5. At 24 h post-infection, RNA was isolated and RT-PCR analysis of HCMV encoded UL40, US11 and US10 was performed. (C) Quantitative RT-PCR analysis of mRNA isolated in B was performed and the ΔΔCT value was determined. Statistical analysis was performed applying a Mann-Whitney U-test. (D) Control HeLa cells (-), US10-expressing HeLa cells, and, in addition, mock treated or infected (24 h, ΔUS2-6, ΔUS2-6/US11, ΔUS2-11; MOI 5) MRC5 cells were metabolically labeled with [35S]-Met/Cys for 2 h and an immunoprecipitation using W6/32 was performed. (E) ARPE19 cells were mock treated or infected with TB40 derived ΔUS2-6 mutant. At 48 h post-infection cells were analysed by flow cytometry as indicated. Lower panel shows the mean regulation of HLA-A*02 and HLA-B*07 by the HCMV mutant compared to mock treated cells with error bars from three independent experiments. Similarly, HLA-A*02 and HLA-B*07 regulation in MRC5 fibroblast by the AD169VarL derived ΔUS2-6 mutant compared to mock treated MRC5 cells is shown (data from experiments also shown in Fig 1D). (TIF) [file ppat.1008040.s001.tif]

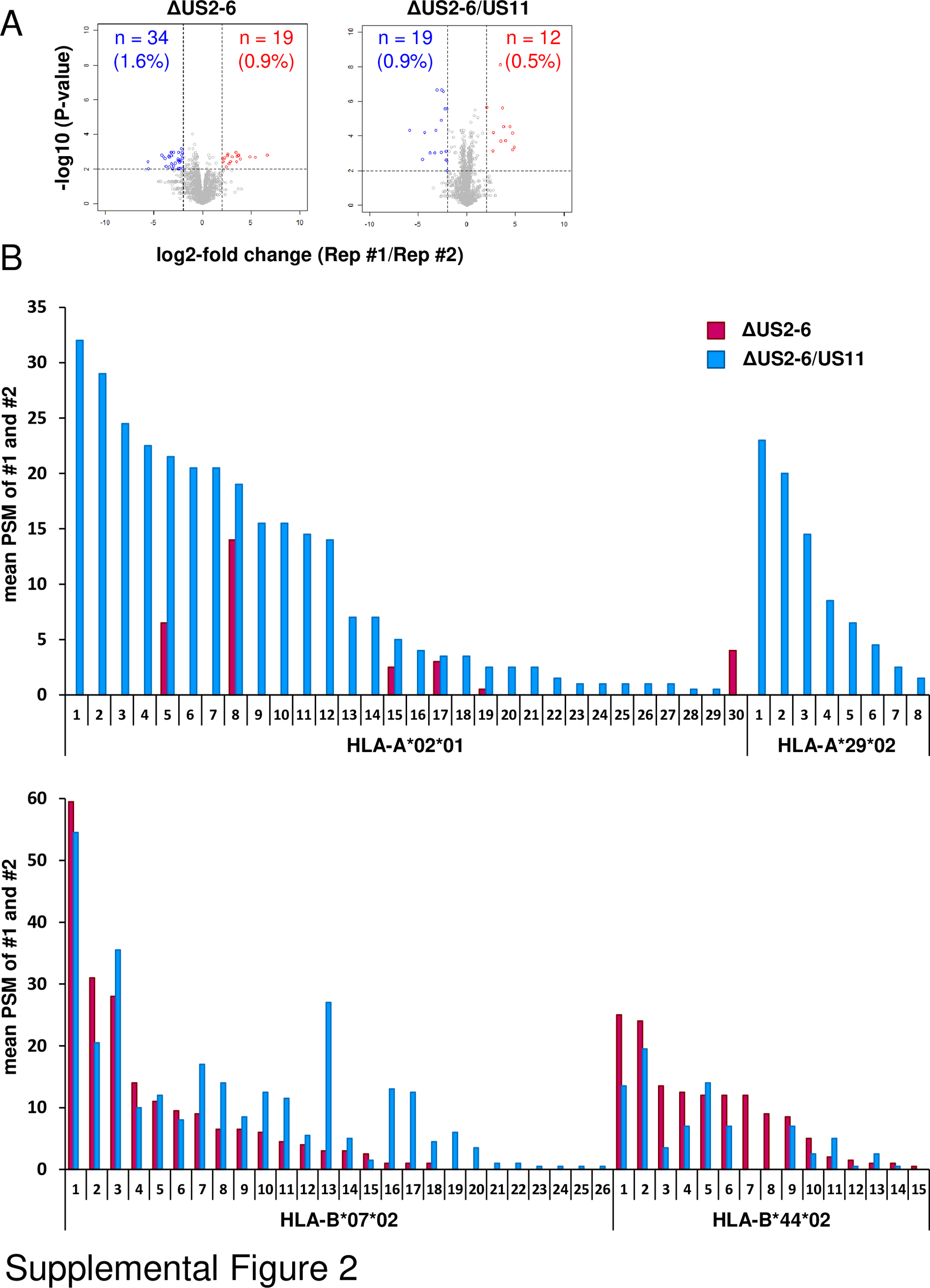

Supplement: S2 Fig — (A) The reproducibility of HLA peptidome analysis is depicted by volcano plots of HLA-I peptide abundances in biological replicates of MRC-5 cells infected with ΔUS2-6 or ΔUS2-6/US11 HCMV mutants shown in Fig 1A and 1B. (B) Depiction of viral peptides (given as numbers on the x-axis) identified in the ligandome analysis from Fig 1A and 1B. The y-axis shows the mean PSM values from two biological replicates. For HLA-A*02:01 and A*29:02 the eluted peptides are ordered according to their abundance in ΔUS2-6 infected cells and for B*07:02 and B*44:02 according to their abundance in ΔUS2-6/US11 infected cells. (TIF) [file ppat.1008040.s002.tif]

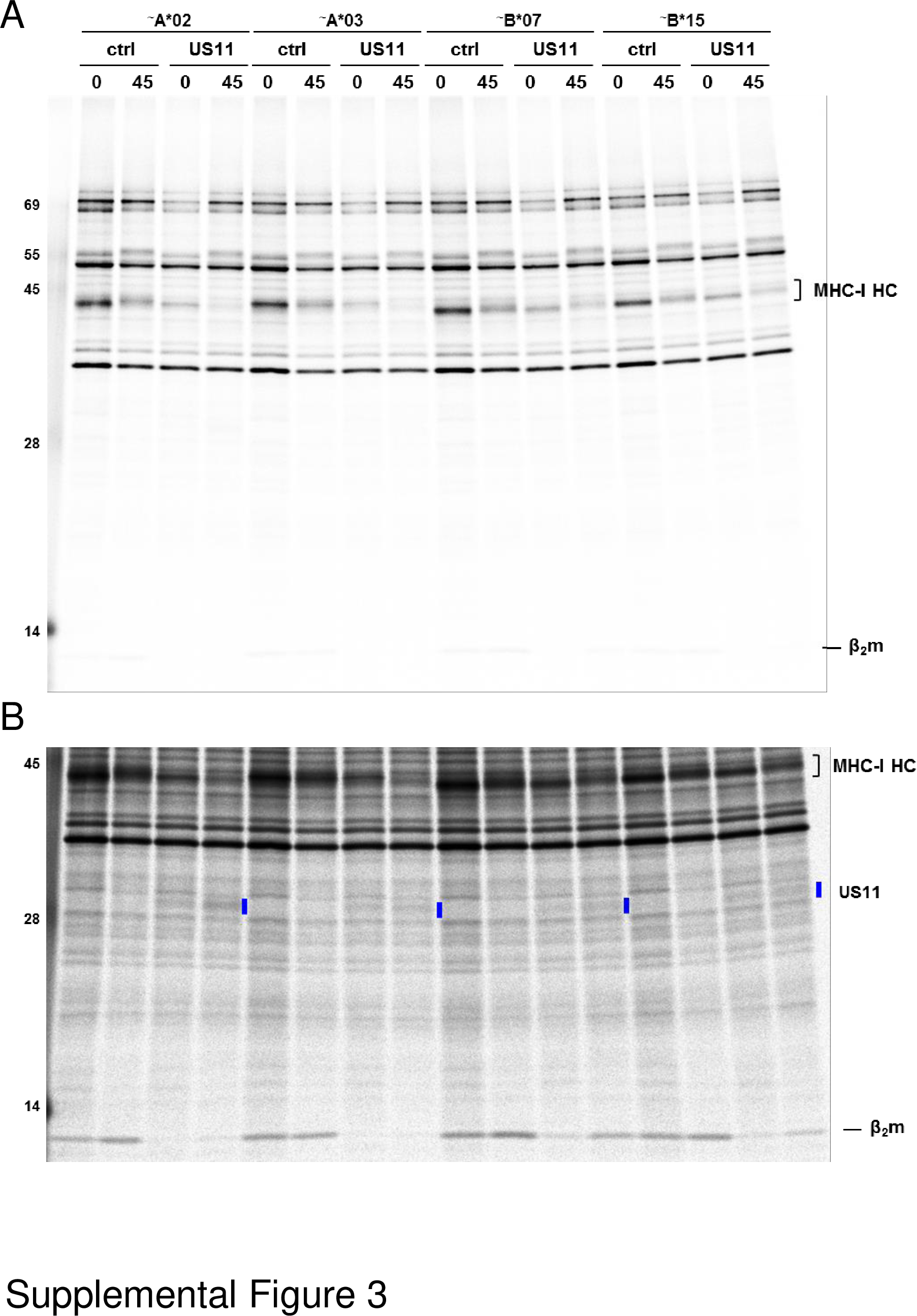

Supplement: S3 Fig — (A) Uncropped gel of results shown in Fig 2C. (B) Gel from A with increased contrast to visualize weak bands. Blue bars indicate a band to the left with the size of US11. (TIF) [file ppat.1008040.s003.tif]

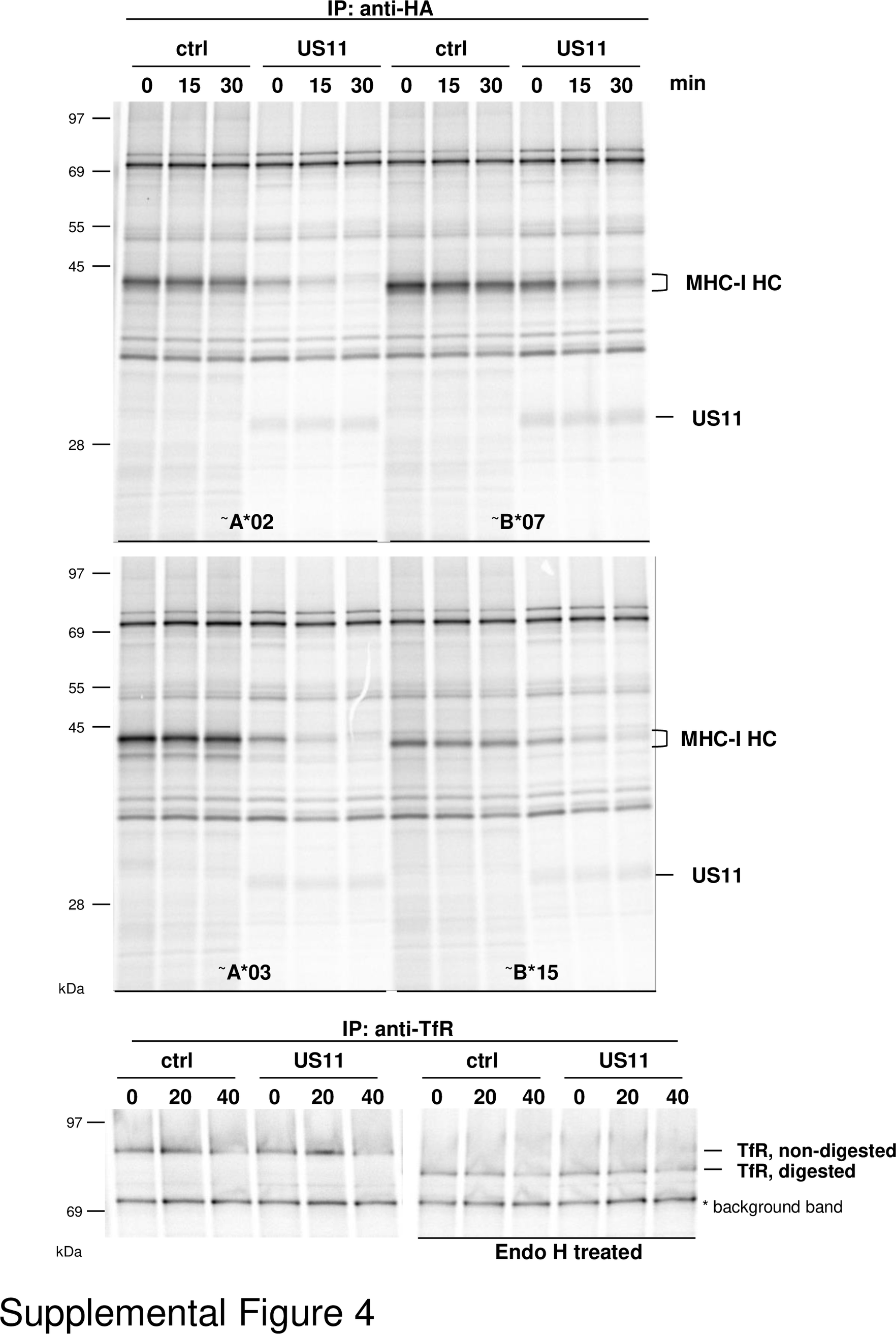

Supplement: S4 Fig — HeLa cells were transiently co-transfected with US11 or a control pIRES-EGFP plasmid (CMV major IE promoter) together with the indicated HA-tagged (~) HLA molecules expressed from the pUC-IP vector (SFFV U3 promoter). At 20 h post-transfection cells were labeled with [35S]-Met/Cys for 15 min and chased for 0, 15 and 30 min and an immunoprecipitation experiment was performed using anti-HA antibodies. The lower panel shows a pulse-chase experiment performed in parallel using anti-TfR mAbs. (TIF) [file ppat.1008040.s004.tif]

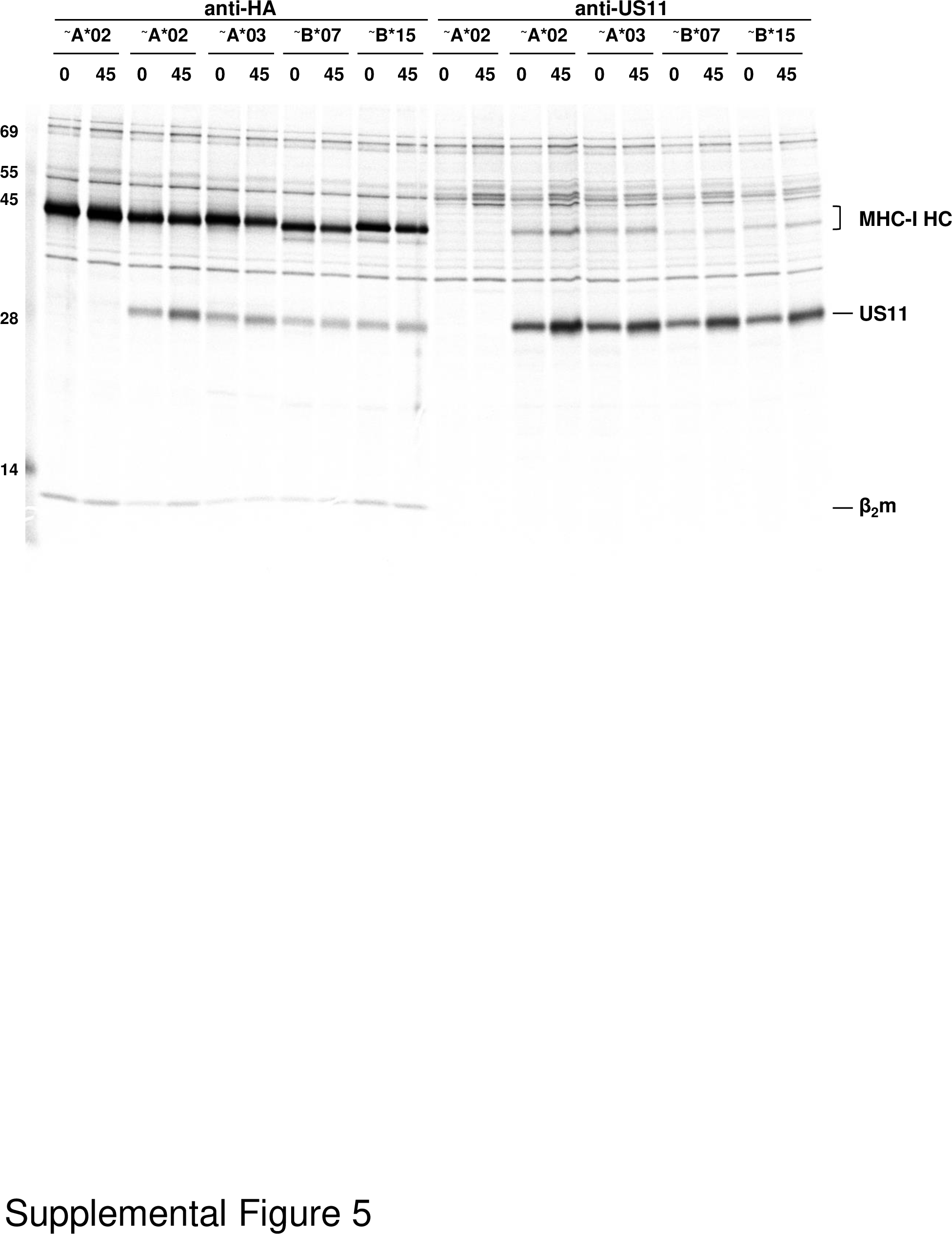

Supplement: S5 Fig — (TIF) [file ppat.1008040.s005.tif]

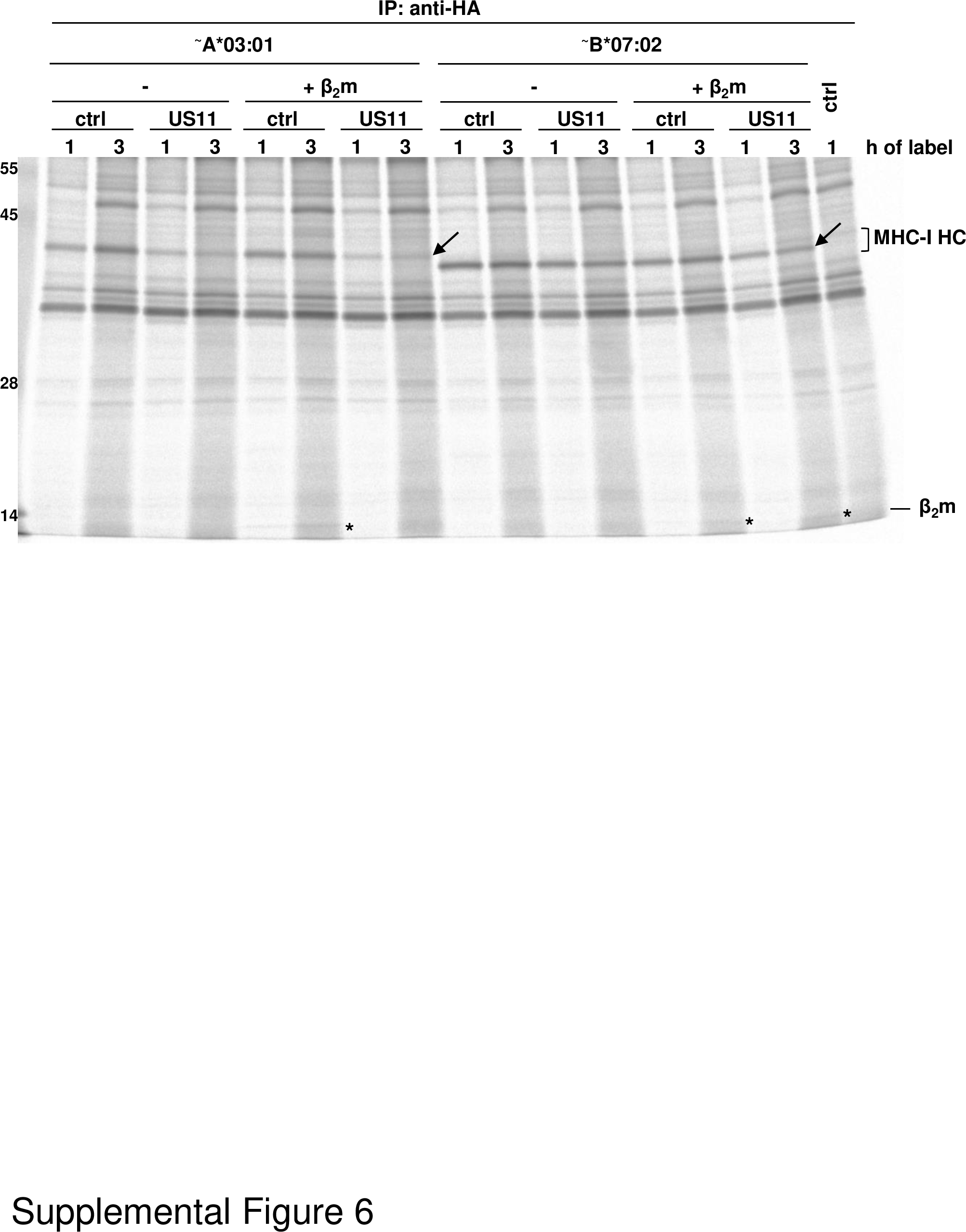

Supplement: S6 Fig — (TIF) [file ppat.1008040.s006.tif]

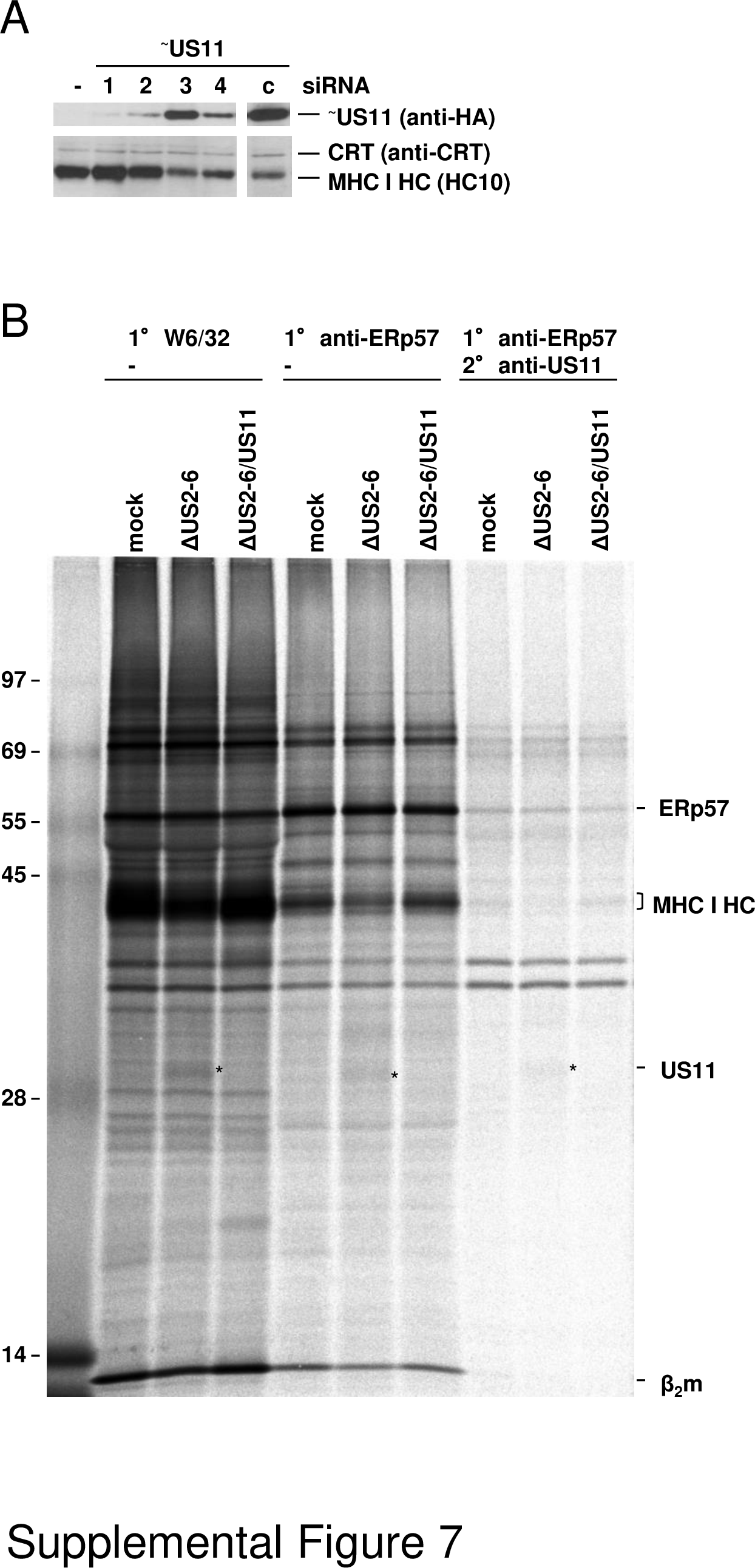

Supplement: S7 Fig — Efficiency of four different siRNAs directed against US11 was tested in HeLa cells stably expressing HA-tagged US11. (A) Western Blot analysis was performed using rabbit anti-HA antibodies, mAb HC10 and as a loading control anti-calreticulin antibodies. Cells were treated with control siRNA (c) or siRNA against US11 (1–4). Control cells without US11 expression and siRNA treatment was included in the analysis (-). US11_1 siRNA was chosen for further experiments. The sequences for the siRNA are: 1, ACACUUGAAUCACUGCCACCCCC; 2, UUGAAUCACUGCCACCAUCCCCC; 3, UCUACAUAAUAAGUUUGGCCCCC; 4, UCGCACUCUACAUAAUAAGCCCCC. (B) Gel shown in Fig 4B, here depicted with same contrast and light settings for all parts. (TIF) [file ppat.1008040.s007.tif]

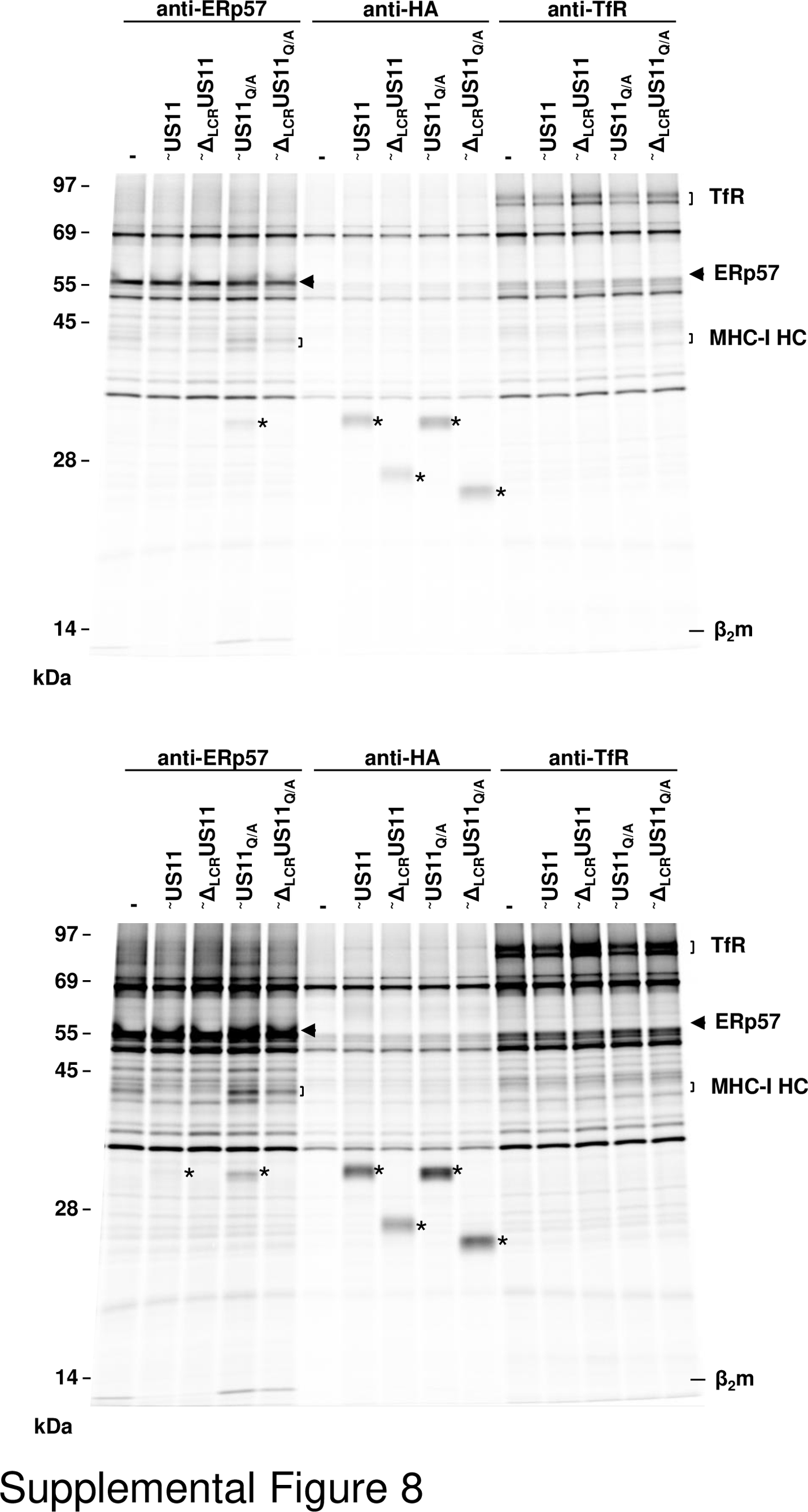

Supplement: S8 Fig — Stably transduced HeLa cells with US11 variants as indicated, were labeled with [35S]-Met/Cys for 2 h and co-immunoprecipitation was performed using antibodies as indicated. Two different contrast and light setting are shown (upper and lower panel). (TIF) [file ppat.1008040.s008.tif]

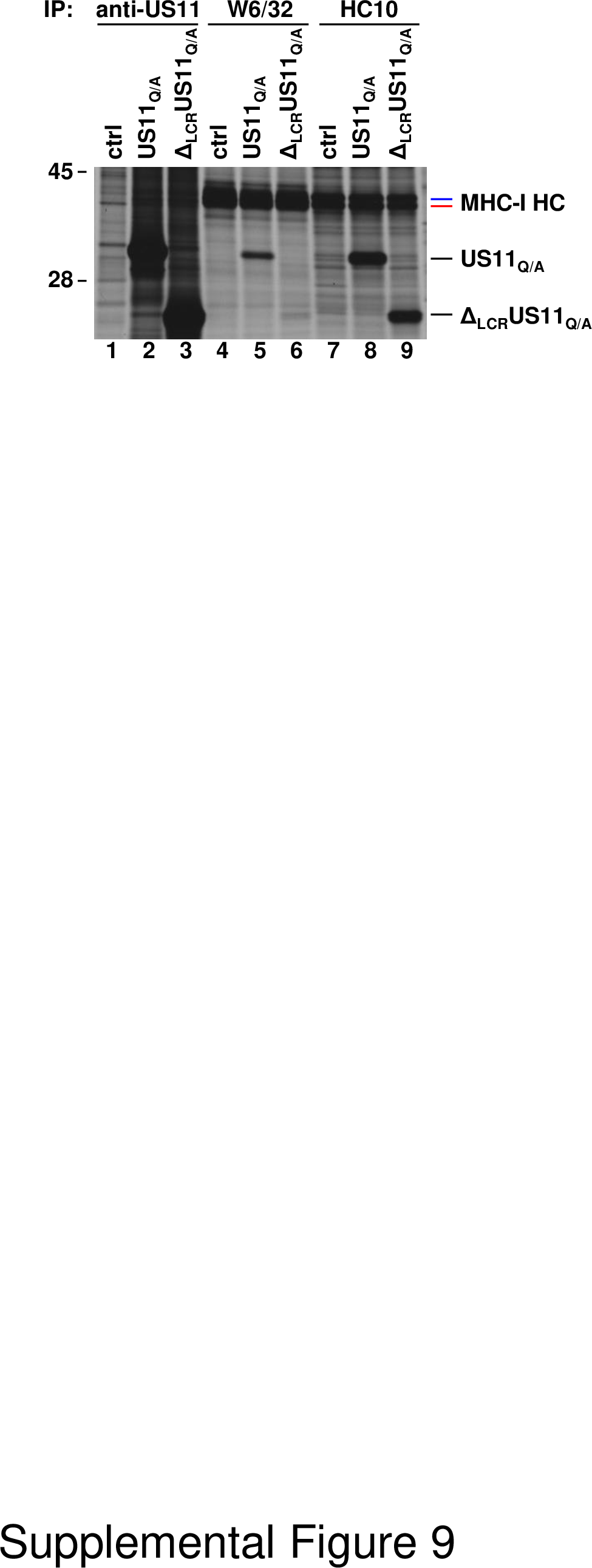

Supplement: S9 Fig — Longer exposure of gel shown in Fig 5E. (TIF) [file ppat.1008040.s009.tif]

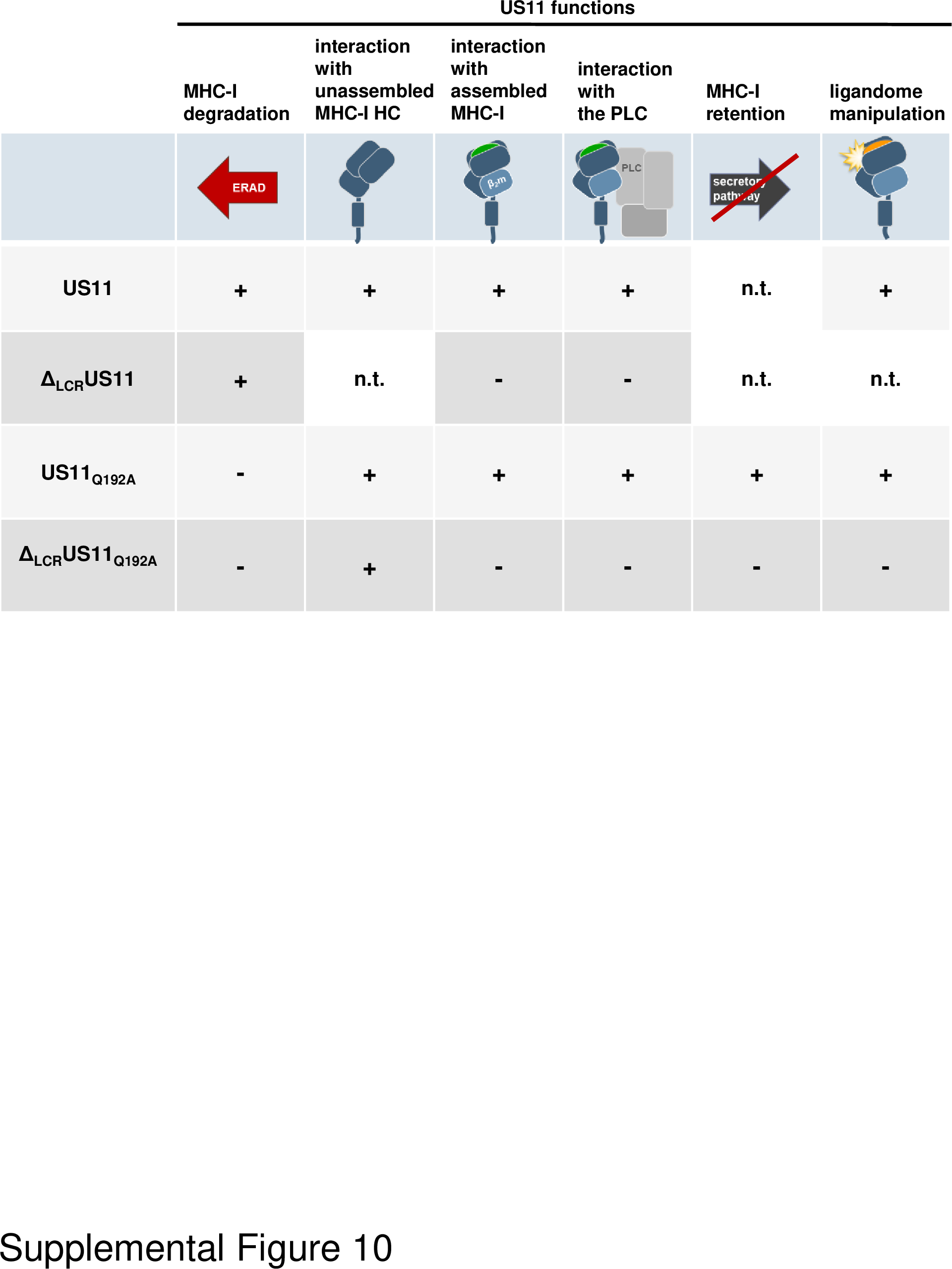

Supplement: S10 Fig — The schematic table depicts effects of the US11 LCR sequence. The table summarizes the findings from the co-immunoprecipitation experiments shown in Fig 5. White cells indicate functions that were not analyzed in detail. In addition, in the last column, also the ability to modify MHC-I peptide loading (results shown in Fig 7) is included. (TIF) [file ppat.1008040.s010.tif]

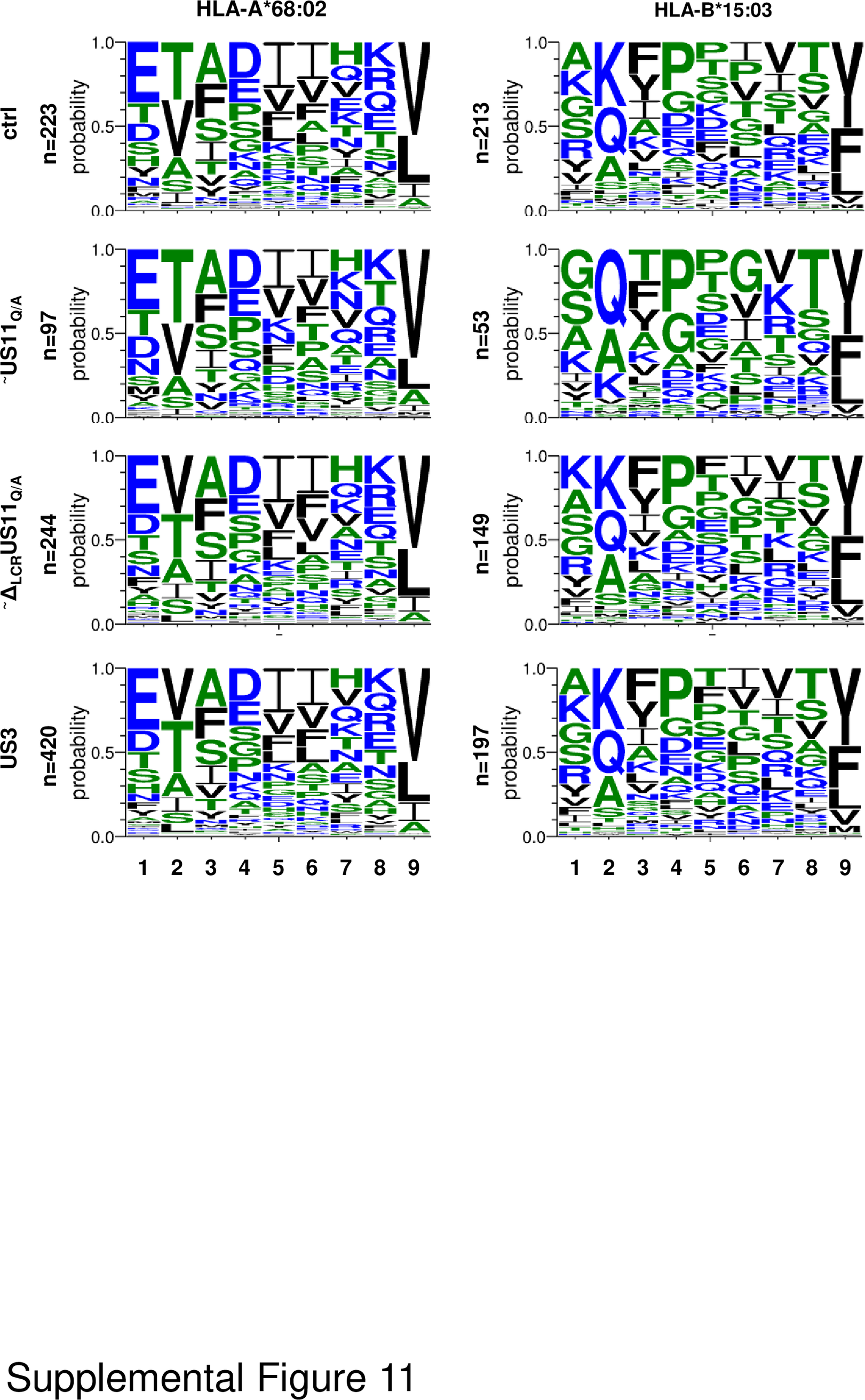

Supplement: S11 Fig — Common HLA-A68:02 and B15:03 9-mer ligands of the biological replicates #1 and #2 (from samples described in Fig 7) are depicted as sequence logos [80]. The numbers below the logos indicate the amino acid position of MHC-I peptide ligands, with HLA-A*68:02 peptide ligands in the left panel and B*15:03 ligands in the right panel. (TIF) [file ppat.1008040.s011.tif]

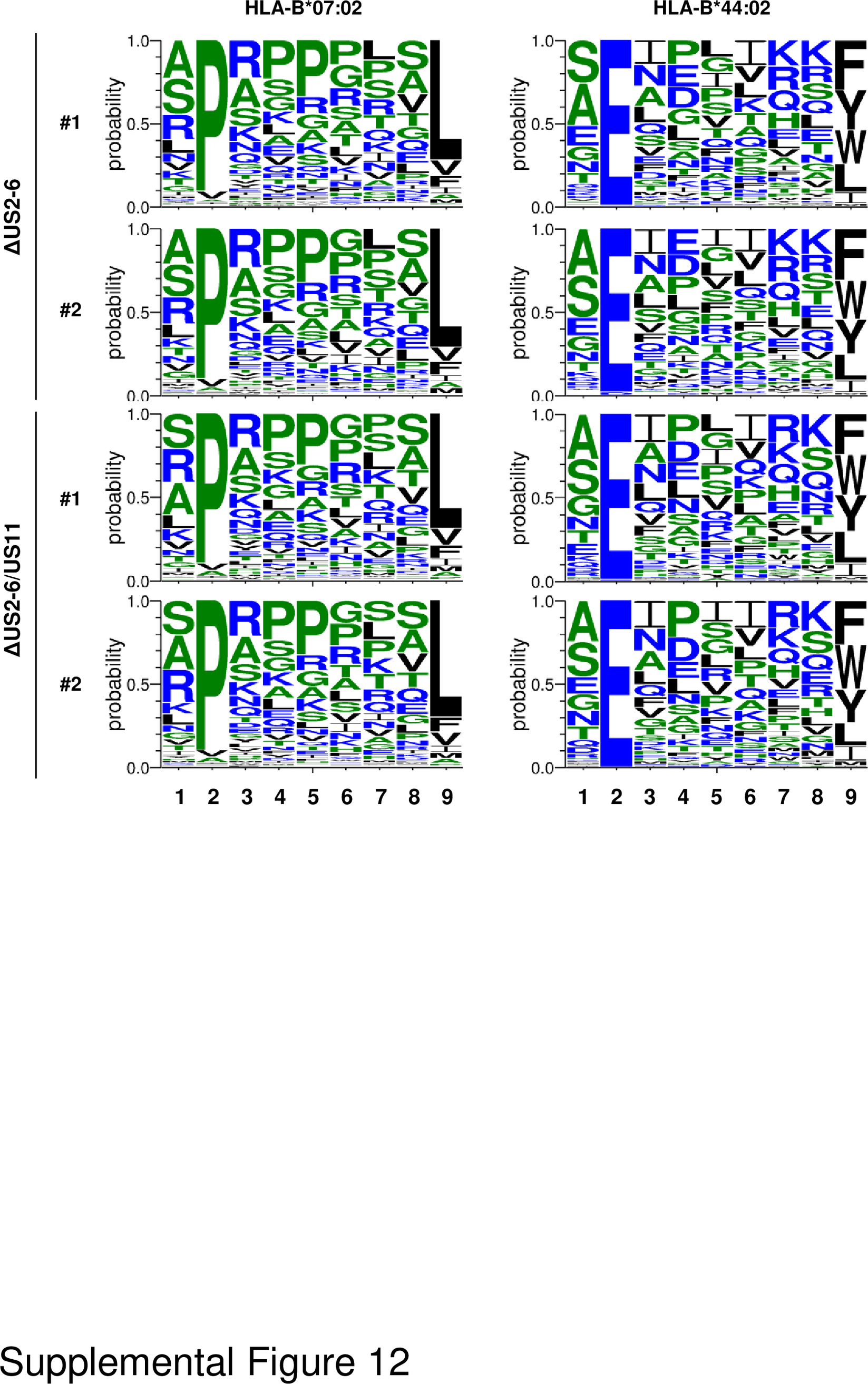

Supplement: S12 Fig — The figure shows sequence logos [80] of the total pool of HLA-B*07:02 and B*44:02 9-mer ligands derived from replicate #1 and #2 depicted in Fig 1. The peptides were considered to be specific ligands if NetMHC3.4 [44] predicted an affinity of <500 and <1000 nM, for HLA-B*07:02 and B*44:02, respectively. The numbers below the logos indicate the amino acid position of MHC-I peptide ligands. (TIF) [file ppat.1008040.s012.tif]

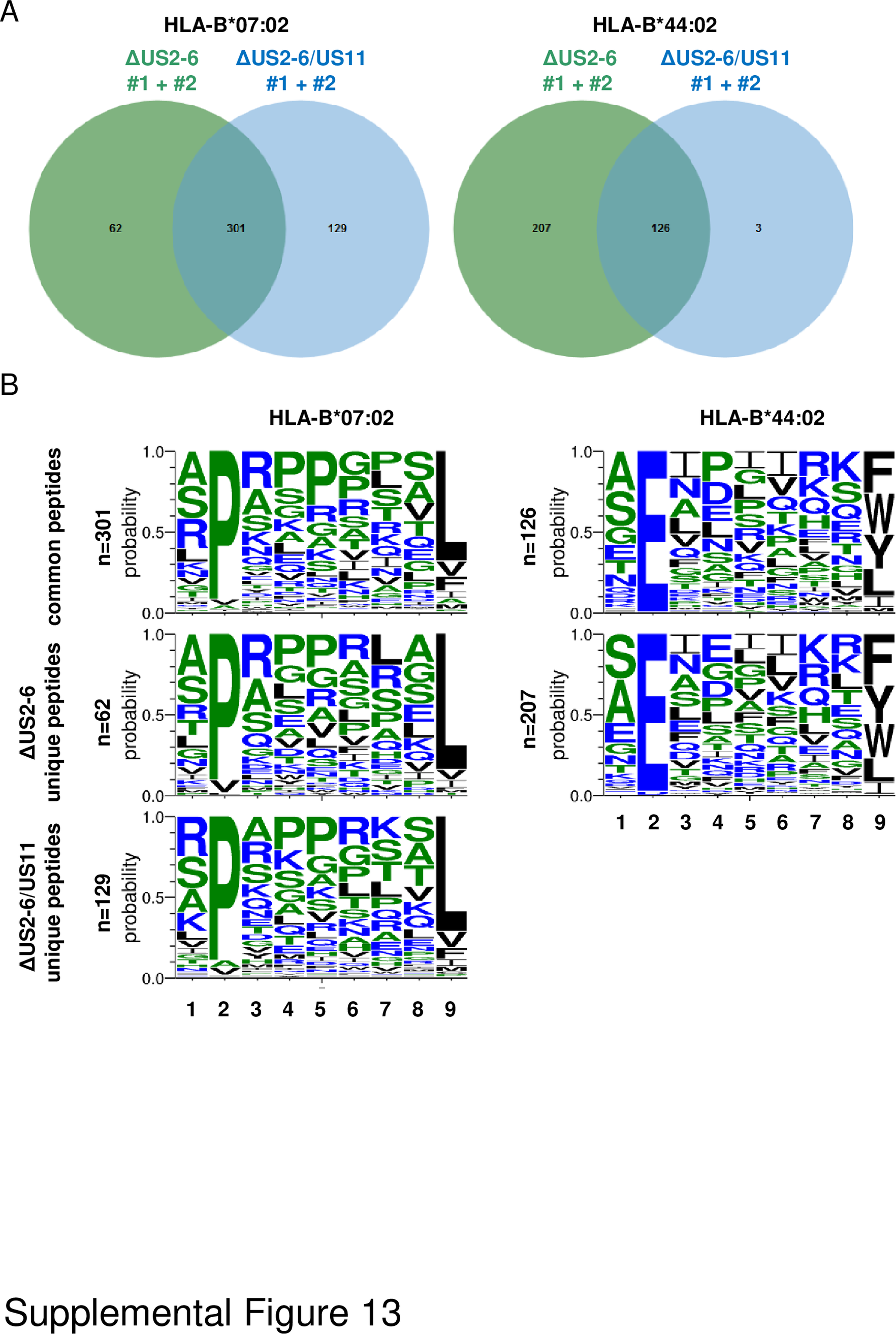

Supplement: S13 Fig — (A) The figure shows the number of common and unique HLA-B ligands from cells infected with ΔUS2-6 and ΔUS2-6/US11 as Venn diagrams [81] (pooled peptides from replicates #1 and #2 as described in S12 Fig). (B) Groups of peptides (common and unique) shown in (A) are depicted as sequence logos [80]. The numbers below the logos indicate the amino acid position of HLA-B peptide ligands. (TIF) [file ppat.1008040.s013.tif]

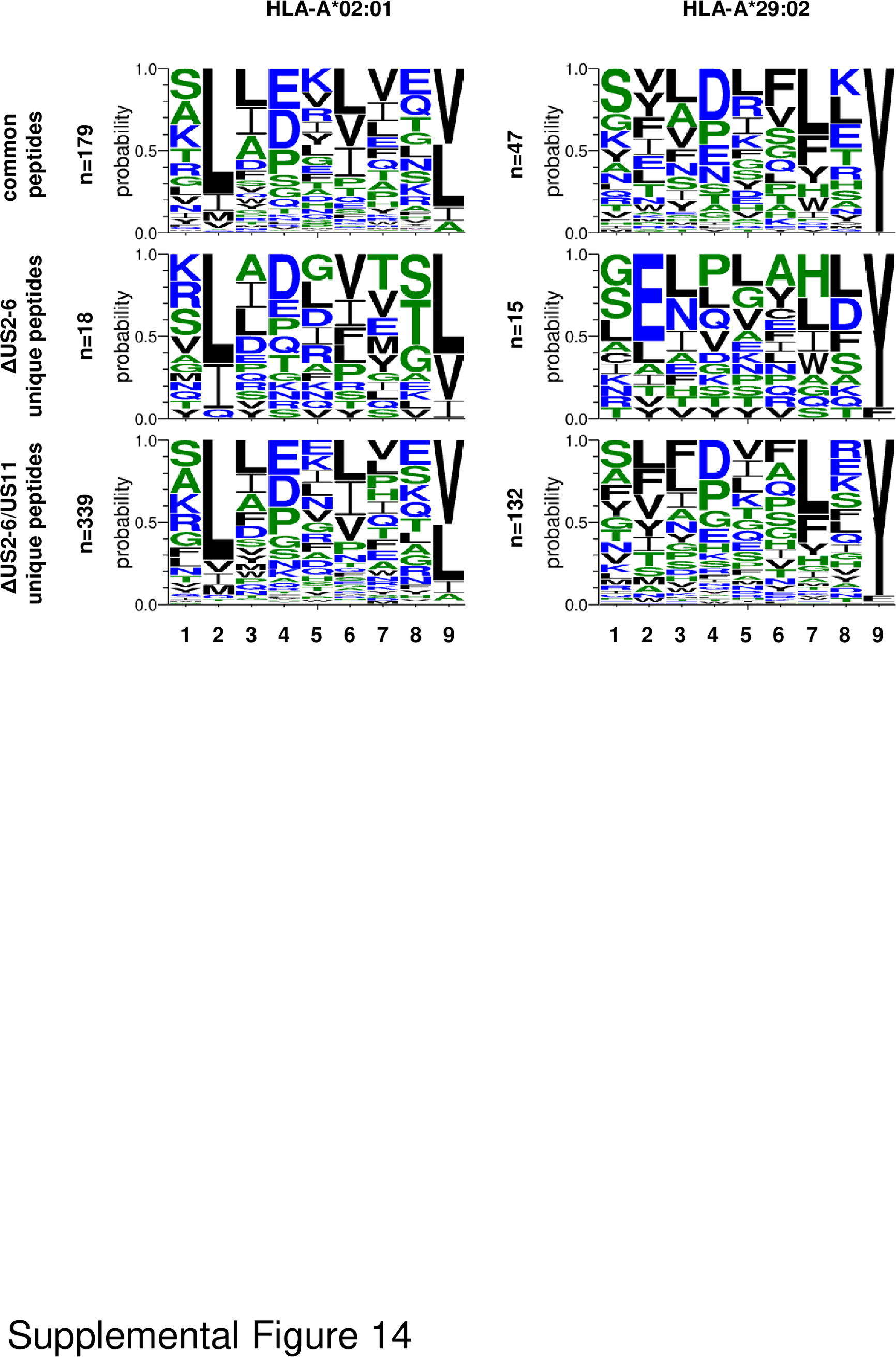

Supplement: S14 Fig — The figure shows common and unique ligands from cells infected with ΔUS2-6 and ΔUS2-6/US11 (pooled peptides from replicates #1 and #2) as sequence logos [80]. The peptides were considered to be specific ligands if NetMHC3.4 [44] predicted an affinity of <500. The number of peptides for each group is given to the left. The numbers below the logos indicate the amino acid position of MHC-I peptide ligands. (TIF) [file ppat.1008040.s014.tif]

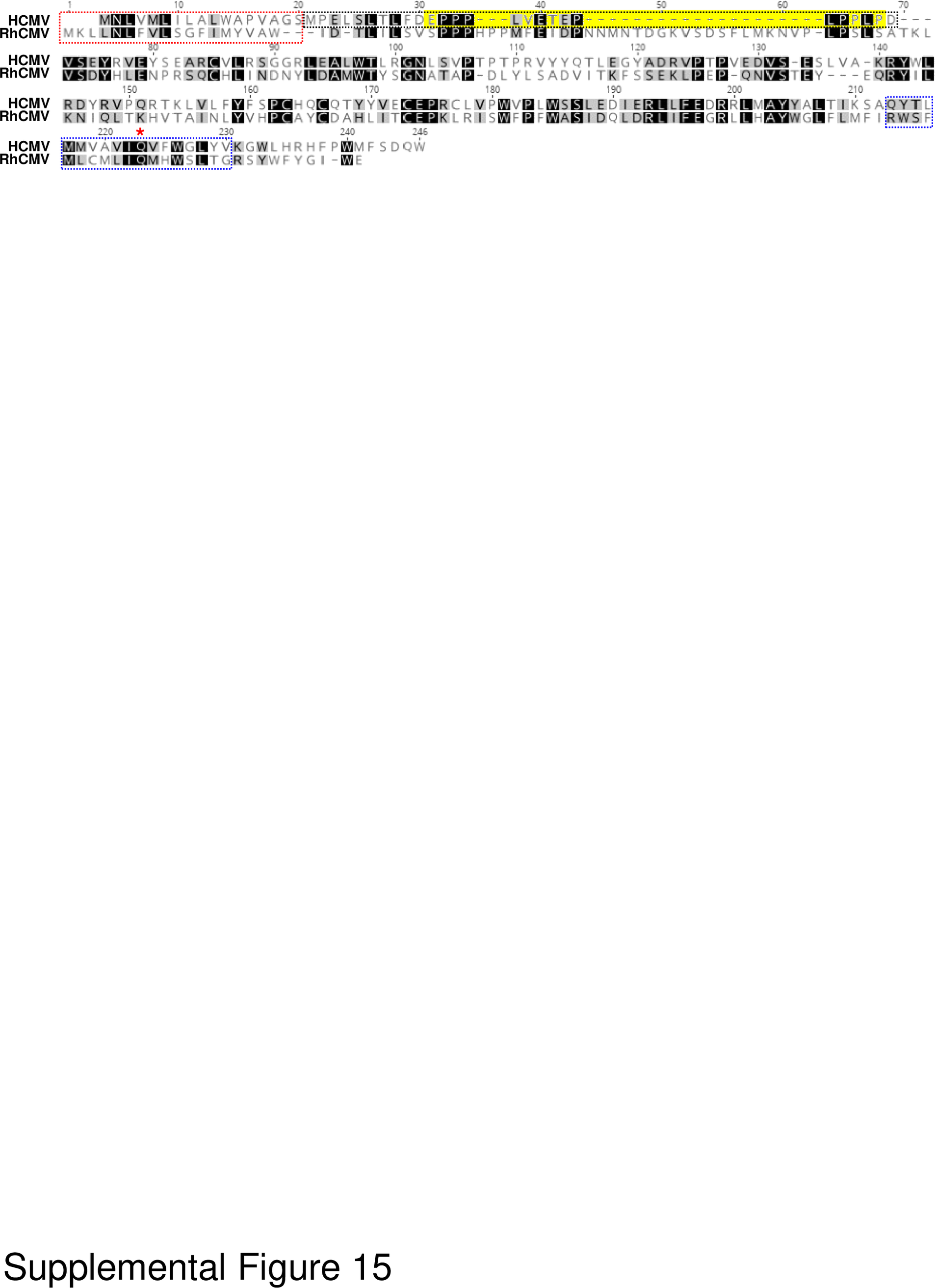

Supplement: S15 Fig — The protein sequence of US11 orthologs is shown. The predicted sequences for the signal peptide is highlighted in red, for the transmembrane segment in blue and the LCR of HCMV encoded US11 in yellow. The N-terminal region deleted in the ΔLCRUS11 mutant is marked with a black box. Highly conserved residues are marked in black, similar residues in grey and non-conserved residues in white. Glutamine 192 of HCMV US11 is marked with a red asterisk. (TIF) [file ppat.1008040.s015.tif]
